# Dynamics of a large multidrug-resistant plasmid encoding New Delhi metallo-β-lactamase-1 and oxacillinase-58 carbapenemases in Acinetobacter baumannii clinical isolates from a tertiary hospital in Malaysia

**DOI:** 10.1099/mgen.0.001630

**Published:** 2026-02-11

**Authors:** Nurul Saidah Din, Farahiyah Mohd Rani, Salwani Ismail, Nor Iza A. Rahman, Hong Leong Cheah, Hock Siew Tan, Sadequr Rahman, David W. Cleary, Qasim Ayub, Stuart C. Clarke, Chew Chieng Yeo

**Affiliations:** 1 Centre for Research in Infectious Diseases and Biotechnology (CeRIDB), Faculty of Medicine, Universiti Sultan Zainal Abidin, Kuala Terengganu, Malaysia; 2 School of Science, Monash University Malaysia, Bandar Sunway, Malaysia; 3 Genomics Platform, Monash University Malaysia, Bandar Sunway, Malaysia; 4 Department of Microbes, Infections and Microbiomes, School of Infection, Inflammation and Immunology, College of Medicine and Health, University of Birmingham, Birmingham, UK; 5 Institute of Microbiology and Infection, University of Birmingham, Birmingham, UK; 6 Faculty of Medicine and Institute for Life Sciences, University of Southampton, Southampton, UK; 7 NIHR Southampton Biomedical Research Centre, University Hospital Southampton Foundation NHS Trust, Southampton, UK; 8 Institute for Research, Development and Innovation, International Medical University, Kuala Lumpur, Malaysia; 9 Department of Biological Sciences, Faculty of Science, Universiti Tunku Abdul Rahman, Kampar, Malaysia

**Keywords:** *Acinetobacter baumannii*, New Delhi metallo-β-lactamase-1 (NDM-1), oxacillinases-58 (OXA-58) carbapenemase, plasmid evolution, transmissible plasmid, *xrs* modules

## Abstract

*Acinetobacter baumannii* carrying the *bla*_NDM-1_ gene, which encodes New Delhi metallo-β-lactamase-1 (NDM-1), exhibits resistance to nearly all β-lactams and is not affected by β-lactamase inhibitors, limiting treatment options. The *bla*_NDM-1_ gene is often associated with other antimicrobial resistance (AMR) genes, resulting in multidrug-resistant (MDR) phenotypes. We previously reported a large, *circa* (ca.) 170 kb plasmid co-harbouring *bla*_NDM-1_- and *bla*_OXA-58_-encoded carbapenemases in clinical MDR *Acinetobacter nosocomialis* and *A. baumannii* isolates from a tertiary hospital in Terengganu, Malaysia, in 2015 and 2016, respectively. In this study, we identified four additional MDR *A. baumannii* isolates from the same hospital (2018–2020) carrying *bla*_NDM-1_ on a similar plasmid. Complete genome sequences were obtained using a hybrid assembly of short-read DNA Nanoball Sequencing(DNBSeq) and long-read (Oxford Nanopore Technologies) data. All isolates belonged to distinct, unrelated clonal lineages, indicating the ongoing horizontal transmission of this plasmid. Comparative analysis revealed substantial structural variability among the plasmids, largely driven by insertion sequence elements and mobile *xrs* (also known as p*dif*) modules. In two *A. baumannii* isolates (i.e. AC1932 and AC2014), *bla*_OXA-58_ and several adjacent *xrs* modules were absent. Despite this, carbapenem minimum inhibitory concentrations remained comparable across all isolates, strongly indicating that *bla*_NDM-1_ is the primary carbapenem resistance determinant. Notably, in *A. baumannii* AC1839, we identified a 15,434 bp presumptive transposon carrying a type III cyclic oligonucleotide-based anti-phage signalling system, inserted within the *xrs*-rich region of the plasmid. AC1839 also carried a separate 11.1 kp plasmid carrying the tetracycline resistance genes *tetA(39)-tetR* within an *xrs* module. Identical *xrs* modules were identified in other unrelated *Acinetobacter* plasmids. These results underscore the mobility and potential roles of *xrs* modules in AMR gene dissemination and, more generally, in shaping *Acinetobacter* plasmid evolution.

**Impact Statement**

Carbapenem-resistant *Acinetobacter baumannii* is listed by the World Health Organization as a top-priority critical pathogen due to its limited treatment options. *A. baumannii* isolates that harbour the *bla*_NDM-1_ gene, which encodes the New Delhi metallo-β-lactamase-1 (NDM-1) enzyme, are not only resistant to carbapenems and β-lactam/β-lactamase inhibitor combinations, they are also multidrug resistant (MDR), posing a significant therapeutic challenge. We previously identified a large, ca. 170 kb plasmid carrying both *bla*_NDM-1_- and *bla*_OXA-58_-encoded carbapenemases, along with multiple antimicrobial resistance genes, in *A. baumannii* and *Acinetobacter nosocomialis* isolates from a tertiary hospital in Terengganu, Malaysia. We have now detected four additional MDR *A. baumannii* isolates from the same hospital carrying similar large plasmids. Complete genome sequencing and analysis revealed that these isolates belonged to distinct clonal lineages, suggesting active horizontal transmission of this plasmid. Structural variations in the plasmid, driven by insertion sequence elements and *xrs* modules, were observed. Notably, the *bla*_OXA-58_ gene was absent in two *A. baumannii* isolates, yet carbapenem resistance persisted, strongly suggesting *bla*_NDM-1_ as the primary driver. Our findings highlight the plasmid’s role in disseminating carbapenem resistance and underscore the urgent need for sustained genomic surveillance of *Acinetobacter* spp. in healthcare settings.

## Data Summary

The authors confirm that all supporting data, code and protocols have been provided within the article or through supplementary data files. The assembled complete genomes in this study have been deposited in the National Center for Biotechnology Information (NCBI) Genomes database under the following accession numbers: *Acinetobacter baumannii* AC1839, JAQIRC000000000; *A. baumannii* AC1932, JAQIQK000000000; *A. baumannii* AC2013, JAQIPS000000000 and *A. baumannii* AC2014, JAQIPR000000000.

## Introduction

For nearly half a century, no new class of antibiotics has been introduced for the treatment of Gram-negative bacteria, including *Acinetobacter baumannii* infections [1,2]. Thus, carbapenem-resistant *A. baumannii* (CRAB) is among the three Gram-negative bacterial pathogens that have received the highest priority classification, and it retained its critical status in the revised 2024 publication of the Bacterial Priority Pathogens List issued by the World Health Organization [3]. Globally, the prevalence of carbapenem resistance among *A. baumannii* has exceeded 90% in some countries, and hospital-acquired pneumonia and bloodstream infections caused by CRAB have been estimated to account for mortality rates of up to 60% [1,4]. During the European Antimicrobial Resistance Surveillance Network (EARS-Net) period, which covered 2017–2021, carbapenem resistance in *Acinetobacter* spp., including CRAB, increased to 74.5% in 2021 compared with 59.2% in 2017 [5], thus highlighting the challenges in combating CRAB infections in the face of increasing resistance [2,6].

The resistance mechanisms employed by CRAB are generally mediated by intrinsic or acquired mechanisms, or a combination of both. A major resistance pathway is the production of β-lactamase enzymes that hydrolyse carbapenems. In *Acinetobacter* spp., the most common carbapenem-inactivating carbapenemases are the Class D [oxacillinases (OXAs)] and Class B [metallo-β-lactamase (MBLs)] β-lactamases [7]. The New Delhi metallo-β-lactamase (NDM) has the ability to hydrolyse almost all β-lactam antibiotics, including carbapenems, and is typically located on conjugative plasmids, facilitating the spread of CRAB infections [8]. NDM-1 is not inhibited by β-lactamase inhibitors such as clavulanic acid, sulbactam, tazobactam and avibactam, thus precluding the use of β-lactam/β-lactamase inhibitor combination therapy [9,10]. In CRAB and other Gram-negative pathogens, the *bla*_NDM-1_ gene is often observed within a Tn*125* composite transposon that is flanked by two copies of the IS*Aba125* insertion sequences (ISs) [11,12] The horizontal transmission of Tn*125* carrying the *bla*_NDM-1_ gene from a CRAB strain to a carbapenem-susceptible *A. baumannii* strain, which was hypothesized to occur by transduction, has been described [13]. More often, *Acinetobacter* spp. harbouring *bla*_NDM-1_ are also multidrug resistant (MDR) due to its association with other antimicrobial resistance (AMR) genes [14,15].

Since its initial spread among the Enterobacterales, *bla*_NDM-1_ was quickly identified in *Acinetobacter* spp., which are considered a key reservoir for the gene [16,17]. NDM-producing *Acinetobacter* spp. have been reported from nearly all continents, often causing difficult-to-treat nosocomial outbreaks [18]. This rapid globalization is driven by the gene’s association with mobile genetic elements, particularly the Tn*125* transposon, which is highly prevalent in *Acinetobacter* genomes [17]. The phenomenon is not limited to *A. baumannii*; the *bla*_NDM-1_ gene has been identified in a wide range of *Acinetobacter* species, including *Acinetobacter nosocomialis*, *Acinetobacter pittii* and *Acinetobacter junnii*, and often on large transmissible plasmids, highlighting its extensive interspecies mobility within the genus [8,16].

This global challenge is reflected at the local level. In Malaysia, NDM-producing *Acinetobacter* have been documented but without any in-depth characterization of their genetic environment [19]. We had reported two *bla*_NDM-1_-producing, carbapenem-resistant MDR *Acinetobacter* spp. isolates from the main tertiary hospital in Terengganu, on the east coast of Peninsular Malaysia: *A. nosocomialis* AC1530, which was isolated in 2015, and *A. baumannii* AC1633, isolated in 2016 [20]. Both isolates harboured an almost identical, ca. 170 kb plasmid (designated pAC1530 and pAC1633-1, respectively) that encoded the *bla*_NDM-1_ gene in Tn*125* and the *bla*_OXA-58_-encoded Class D carbapenemase located within a mobile Xer recombination site (*xrs*) or plasmid-*dif* (p*dif*) module. These modules form a novel class of discrete mobile elements that mobilize via Xer-mediated site-specific recombination through a cointegration/resolution mechanism [21,22]. Since the discovery of the *dif* module, which carried *bla*_OXA-24_ in the *A. baumannii* plasmid pABVA01 [23], these elements have been identified in numerous *Acinetobacter* plasmids. They are characterized by flanking XerC/XerD recombination sites and were initially designated p*dif* (as well as Re27 and pXerC/XerD) to differentiate them from chromosomal *dif* sites [24,25]. However, these elements have been recently redesignated *xrs* for ‘Xer recombination sites’ [18] since ‘*dif*’ is an acronym for ‘deletion-induced filamentation site’, which is a phenotype not associated with plasmid *xrs* sites.

Plasmids pAC1530 and pAC1633-1 also co-located numerous other AMR genes [20]. The discovery of this plasmid in two different species of *Acinetobacter* from the same hospital strongly suggested horizontal transmission, but conventional conjugative assays failed to detect such transfer [20]. Further surveillance from 2018 to 2020 identified four additional *bla*_NDM-1_-carrying CRAB isolates, two of which also co-harboured *bla*_OXA-58_ [26]. Since these isolates were initially sequenced using only short-read technology, determining the complete structure of the plasmids and confirming their relationship to the original pAC1530/pAC1633-1 was challenging.

In this study, the genomes of these four recent *bla*_NDM-1_-carrying *A. baumannii* isolates were subjected to long-read Nanopore sequencing and hybrid assembly with previous short-read data. The aim was to obtain their complete genome sequences, fully resolve the structures of their large *bla*_NDM-1_ plasmids and investigate the structural dynamics of this plasmid across different *Acinetobacter* genomes over a 6-year period (2015–2020).

## Methodology

### Bacterial collection and antibiotic susceptibility tests

Four clinical isolates of *bla*_NDM-1_-positive *A. baumannii* investigated in this study were collected between 2018 and 2020 at Hospital Sultanah Nur Zahirah (HSNZ), the main tertiary hospital in Terengganu, Malaysia. These isolates were among the 126 Terengganu *A. baumannii* isolates whose genomes were recently published [26].

Antimicrobial susceptibility testing was performed using the BD Phoenix™ M50 System (Becton, Dickinson and Company, USA) with the BD Phoenix NMIC-500 panel (Becton, Dickinson and Company, USA). The susceptibility criteria were determined following the latest Clinical and Laboratory Standards Institute guidelines (M100-S35) [27]. CRAB isolates exhibited resistance to imipenem and meropenem. The NMIC-500 panel that was used also detected and classified carbapenemase-producing organisms (CPOs) [28].

### Whole-genome sequencing and sequence analysis

Bacterial genomes were extracted using the GenJET Genomic DNA Purification kit (Thermo Scientific, MA, USA) for short-read sequencing and a conventional phenol–chloroform protocol [29] for extracting high-molecular-weight genomic DNA for long-read sequencing. The DNA was subjected to whole-genome sequencing on the DNBSEQ (MGI Tech Co. Ltd., Shenzhen, China) and the Nanopore MinION (Oxford Nanopore Technologies, Oxford, UK) sequencing platforms. Paired-end DNA libraries were constructed with an average insert size of 100 bp for short-read sequencing using the DNBSEQ-G400 by a commercial service provider (BGI, Shenzhen, China). For long-read sequencing, DNA libraries were constructed using the Rapid Barcoding kit (SQK- RBK114.24) and sequenced using R10.4 flow cells on a MinION device. Basecalling was performed using MinKNOW in super-accuracy basecalling mode. FastQC (http://www.bioinformatics.babraham.ac.uk/projects/fastqc/) was used for quality control of raw reads. A hybrid assembly was then performed using Unicycler v0.5.1 (https://github.com/rrwick/Unicycler) [30], incorporating both the short paired-end reads and the long reads. The assembly quality was checked using QUAST v5.3.0 (https://github.com/ablab/quast).

Genome annotation was performed using PROKKA (https://github.com/tseemann/prokka), and the generated General Feature Format (GFF) file was then used in Roary (https://github.com/tseemann/prokka) to obtain the core-genome alignment using the criteria of 95% sequence identities and presence in 99% of genomes [20,31]. The list of *A. baumannii* genomes used to construct the core-genome alignment is provided in Table S1, available in the online Supplementary Material. VeryFastTree (https://github.com/citiususc/veryfasttree) was used to construct the maximum-likelihood phylogenetic tree with 1,000 bootstrap replicates under the generalized time-reversible model [32]. The phylogenetic tree was then visualized using iTOL v6 (https://itol.embl.de/) [33]. Multilocus sequence typing (MLST) profiles were analysed using the MLST Oxford and Pasteur scheme via PubMLST (https://pubmlst.org/organisms/acinetobacter-baumannii) [34]. The capsular (K) and outer core (OC) loci were identified using Kaptive (https://kaptive-web.erc.monash.edu/) [35] with default parameters. ResFinder (http://genepi.food.dtu.dk/resfinder) [36] and CARD (https://card.mcmaster.ca/) [37] were used to screen for the presence of antibiotic resistance genes. IS elements were determined using ISfinder (https://isfinder.biotoul.fr/) [38], while the presence of *xrs* (or p*dif*) sites was determined by a combination of p*dif* finder (https://github.com/mjshao06/pdifFinder) [39] and manual blastn screening using known XerC/XerD and XerD/XerC sites in the pAC1530 and pAC1633-1 plasmids. Analysis of SNPs in the core regions of the plasmids was performed using snp-dists v0.8.2 (https://github.com/tseeman/snp-dists), with pAC1530 as the reference plasmid. The SNP-based phylogenetic tree of the *bla*_NDM-1_-encoded plasmids was generated using RAxML-NG (https://github.com/amkozlov/raxml-ng) and visualized with iTOL v6. Phage defence genes were identified using DefenseFinder (https://defensefinder.mdmlab.fr/) [40]. Comparative genome analysis was carried out using the National Center for Biotechnology Information (NCBI) blast (https://blast.ncbi.nlm.nih.gov/Blast.cgi) between the current plasmids and the reference genomes, and these were then visualized using EasyFig v2.2.5 (https://mjsull.github.io/Easyfig/) [41].

## Results

### Characteristics of CRAB clinical isolates from HSNZ harbouring *bla*_NDM-1_

Genome analyses of the 126 *A. baumannii* clinical isolates obtained over a 10-year period (2011–2020) from HSNZ led to the identification of five CRAB isolates harbouring the *bla*_NDM-1_ gene (AC1633, AC1839, AC1932, AC2013 and AC2014) [26]. The complete genome sequences of *A. baumannii* AC1633, which was isolated in 2016, had previously been determined together with AC1530, a *bla*_NDM-1_-carrying, carbapenem-resistant *A. nosocomialis* that was obtained from the same hospital in 2015 [20]. These two isolates were the earliest *Acinetobacter* spp. in our collection found to harbour an almost identical, ca. 170 kb plasmid (designated pAC1633-1 in *A. baumannii* AC1633; accession no. CP059301, and pAC1530 in *A. nosocomialis* AC1530; accession no. CP045561) that encodes the *bla*_NDM-1_ gene along with the *bla*_OXA-58_ carbapenemase [20]. Both plasmid sequences were used as reference sequences for this study.

Of the four remaining *bla*_NDM-1_-positive *A. baumannii* isolates, *A. baumannii* AC1839 was recovered from a urine culture of a 1-year-old male patient in the paediatric unit of HSNZ in April 2018, while *A. baumannii* AC1932 was isolated from a blood culture of a 50-year-old female patient in the renal care unit in March 2019. *A. baumannii* AC2013 and *A. baumannii* AC2014 were both obtained from blood cultures of two different female patients (aged 69 and 45, respectively) on the same day (16 February 2020) in the same medical ward, 8C (Table 1). These four *A. baumannii* isolates were phenotypically resistant to all β-lactam antibiotics tested, including cephalosporins and carbapenems [minimum inhibitory concentrations (MICs) of imipenem ≥8 µg ml^−1^, meropenem ≥32 µg ml^−1^ and ertapenem ≥1 µg ml^−1^] (Table 2), but were susceptible to ciprofloxacin, levofloxacin, minocycline, tigecycline and colistin. Among the aminoglycosides, all four isolates showed resistance to gentamicin but were susceptible to amikacin (Table 2). However, only *A. baumannii* AC1839 was resistant to tetracycline among the four isolates, as shown by previous disc diffusion assays [26]. All four isolates were also resistant to trimethoprim–sulphamethoxazole (Table 2). Based on the BD Phoenix™ M50 System, the four *A. baumannii* isolates were determined to be Class B CPOs.

**Table 1.** Background and genomic characteristics of the four *bla*_NDM-1_-positive *A. baumannii* isolates from HSNZ, Terengganu

Isolate	Year of isolation/ward	Specimen	Oxford ST	Pasteur ST	Chromosome/ plasmid	Size (bp)	G+C content (mol%)	AMR gene	Plasmid *rep* type*
AC1839	2 April 2018/Paediatrics	Urine	3348	2575	Chromosome	3,830,773	39.08	*bla*_ADC-270_, *bla*_OXA-65_, *ant(3")-IIc*	
					pAC1839-1	190,660	38.41	*bla*_NDM-1_ *, bla*_OXA-58_ *, aac(3)-IId, aph(3")-Ib, aph(6)-Id, sul2, msrE, mphE*	nd
					pAC1839-2	11,127	33.62	*tetA(39)*	Rep_3 T13
AC1932	7 March 2019/Renal Ward	Blood	1496	1405	Chromosome	3,857,525	39.13	*bla*_ADC-32_, *bla*_OXA-120_, *ant(3")-IIc*	
					pAC1932-1	164,059	38.48	*bla*_NDM-1_, *aac(3)-IId*, *aph(3")-Ib*, *aph(6)-Id*, *sul2*, *msrE*, *mphE*	nd
					pAC1932-2	96,136	40.26	nd	Rep_3 T7
					pAC1932-3	35,240	39.38	nd	nd
AC2013	16 February 2020/Medical Ward 8C	Blood	942	267	Chromosome	3,644,752	38.98	*bla*_ADC-291_, *bla*_OXA-180_, *ant(3")-IIc*	
					pAC2013-1	170,902	38.32	*bla*_NDM-1_, *bla*_OXA-58_, *aac(3)-IId*, *aph(3")-Ib, aph(6)-Id, sul2, msrE, mphE*	nd
					pAC2013-2	7145	31.99	nd	Rep_3 T5
					pAC2013-3	5139	36.56	nd	Rep_3 T79
AC2014	16 February 2020/Medical Ward 8C	Blood	3393	142	Chromosome	3,765,139	39.06	*bla*_ADC-238_, *bla*_OXA-510_, *ant(3")-IIc*	
					pAC2014-1	166,923	38.53	*bla*_NDM-1_, *aac(3)-IId*, *aph(3")-Ib*, *aph(6)-Id*, *sul2*, *msrE*, *mphE*	nd
					pAC2014-2	95,694	38.42	nd	Rep_3_T21
					pAC2014-3	40,116	36.35	nd	Rep_3 T27
					pAC2014-4	5304	32.77	nd	Rep_3 T13

*Plasmid *rep* type was determined using the database described in [34] and [35].

nd, not detected.

**Table 2.** AMR profiles of the *bla*_NDM-1_-positive *A. baumannii* isolates from HSNZ, Terengganu

Antimicrobial class	Antimicrobial compound	AC1633		AC1839		AC1932		AC2013		AC2014	
		MIC*	Interpretation†	MIC*	Interpretation†	MIC*	Interpretation†	MIC*	Interpretation†	MIC*	Interpretation†
Aminoglycosides	AMK	≤4	S	≤4	S	≤4	S	8	S	≤4	S
	GEN	>8	R	>8	R	>8	R	>8	R	>8	R
Carbapenems	ETP	>1	R	>1	R	>1	R	>1	R	>1	R
	IPM	>8	R	>8	R	>8	R	>8	R	>8	R
	MEM	>32	R	>32	R	>32	R	>32	R	>32	R
Cephalosporins	CZO	>16	R	>16	R	>16	R	>16	R	>16	R
	FEP	>16	R	>16	R	>16	R	>16	R	>16	R
	FOX	>16	R	>16	R	>16	R	>16	R	>16	R
	CAZ	>16	R	>16	R	>16	R	>16	R	>16	R
	CRO	>4	R	>4	R	>4	R	>4	R	>4	R
	CXM	>16	R	>16	R	>16	R	>16	R	>16	R
Cephalosporins + β-lactamase Inhibitors	CZA	>8/4	R	>8/4	R	>8/4	R	>8/4	R	>8/4	R
Fluoroquinolones	CIP	>2	R	0.25	S	≤0.125	S	0.5	S	≤0.125	S
	LVX	4	I	≤1	S	≤1	S	≤1	S	2	S
	NOR	>8	I	4	S	≤2	S	4	S	≤2	S
Monobactams	ATM	>16	R	>16	R	>16	R	>16	R	>16	R
Nitrofurans	NIT	>128	R	>128	R	>128	R	>128	R	>128	R
Penicillins	AMP	>16	R	>16	R	>16	R	>16	R	>16	R
Penicillins + β-lactamase Inhibitors	SAM	>16/8	R	>16/8	R	>16/8	R	>16/8	R	>16/8	R
	TZP	>64/4	R	>64/4	R	>64/4	R	>64/4	R	>64/4	R
Phosphonic acid derivatives	FOS	>128	R	>128	R	>128	R	>128	R	>128	R
Polymyxins	COL	≤1	S	≤1	S	≤1	S	≤1	S	≤1	S
Sulphonamides	SXT	>2/38	R	>2/38	R	>2/38	R	>2/38	R	>2/38	R
Tetracyclines	MNO	≤1	S	≤1	S	≤1	S	≤1	S	≤1	S
	TGC	≤1	S	≤1	S	≤1	S	≤1	S	≤1	S

*MIC values are presented as µg ml−1. †I, intermediate; R, resistant; S, susceptible; interpreted according to the latest Clinical and Laboratory Standards Institute guidelines (M100-S35) [27].

AMK, amikacin; AMP, ampicillin; ATM, aztreonam; CAZ, ceftazidime; CIP, ciprofloxacin; COL, colistin; CRO, ceftriaxone; CXM, cefuroxime; CZA, ceftazidime–avibactam; CZO, cefazolin; ETP, ertapenem; FEP, cefepime; FOS, fosfomycin; FOX, cefoxitin; GEN, gentamicin; IPM, imipenem; LVX, levofloxacin; MEM, meropenem; MNO, minocycline; NIT, nitrofurantoin; NOR, norfloxacin; SAM, ampicillin–sulbactam; SXT, trimethoprim–sulphamethoxazole; TGC, tigecycline; TZP, piperacillin–tazobactam.

### Genome characteristics of the *bla*_NDM-1_-positive *A. baumannii* isolates

Hybrid assembly of DNBSeq paired-end short-read and Nanopore long-read sequence data led to the generation of the complete genome sequences of the four *bla*_NDM-1_-positive *A. baumannii* isolates. Each isolate comprised a circular chromosome of ca. 3.64–3.85 Mbp with highly similar mol% G+C content and plasmids of varying sizes (Table 1). MLST analysis of the *bla*_NDM-1_-positive *A. baumannii* genomes revealed that they were non-clonal and belonged to four distinct Oxford and Pasteur Sequence Types (STs) [26] (Table 1). *A. baumannii* AC1839 belonged to novel STs for both the Oxford (assigned by PubMLST curators as ST3348: *gltA*-1, *gyrB*-1, *gdhB*-80, *recA*-6, *cpn60*-42, *gpi*-110, *rpoD*-26) and Pasteur schemes (assigned by PubMLST as ST2575: *cpn60*-56, *fusA*-100, *gtlA*-2, *pyrG*-2, *recA*-9, *rplB*-4, *rpoB*-5) (Table 1). AC2014 belonged to a pre-existing ST142_Pasteur_ but a novel Oxford ST (assigned as ST3393: *gltA*-36, *gyrB*-12, *gdhB*-62, *recA*-31, *cpn60*-22, *gpi*-534, *rpoD*-4). *A. baumannii* AC2013, which was isolated on the same day and from the same ward, albeit from different patients, as AC2014, was ST942_Oxford_ and ST267_Pasteur_ (Table 1). Core genome phylogenetic analysis of Malaysian *A. baumannii* isolates, alongside several reference genomes, clearly demonstrated that the *bla*_NDM-1_-carrying isolates were genetically distinct and unrelated (Fig. S1).

These *A. baumannii* isolates harboured a large, *bla*_NDM-1_-encoding plasmid ranging in size between 164,059 and 190,660 bp, with two of these plasmids (namely pAC1839-1 and pAC2013-1 in *A. baumannii* AC1839 and AC2013, respectively) harbouring both *bla*_NDM-1_ and *bla*_OXA-58_ (Table 1). None of these four large plasmids carried any known plasmid replication initiator (or *rep*) gene that is found in the Acinetobacter Plasmid Typing database [42,43], similar to what was observed for pAC1633-1 isolated from *A. baumannii* AC1633 and pAC1530 from *A. nosocomialis* AC1530 [20]. This is suggestive of a novel *rep* gene encoded by these plasmids that has yet to be characterized. These plasmids harbouring *bla*_NDM-1_ currently have no homologous counterparts in the databases and are, therefore, unique to *Acinetobacter* isolates from Malaysia. Of note, we had postulated that pAC1530 and pAC1633-1 were derived from an IS*1006*-mediated cointegration of two plasmids – one carrying *bla*_NDM-1_ and the other encoding *bla*_OXA-58_ – due to the high sequence identity with the two plasmids found in *A. pittii* AP882, a hospital isolate from the state of Perak, Malaysia, in 2014 [20,44]. A blastn comparison of recently reported large *Acinetobacter* plasmids harbouring *bla*_NDM-1_ and *bla*_OXA-58_ [15] showed sequence similarities only at the Tn*125* region that contained *bla*_NDM-1_ and in the sequences surrounding the *bla*_OXA-58_ region (Fig. S2), thus reinforcing the uniqueness of the plasmid backbone harbouring these two carbapenemase genes from Malaysia. Likewise, blastn with the NCBI non-redundant database did not result in any significant hits beyond the Tn*125*-*bla*_NDM-1_ and *bla*_OXA-58_ regions.

The four *bla*_NDM-1_-positive *A. baumannii* isolates also harboured an additional one to three other plasmids that ranged in size from 5,139 to 96,136 bp, most of which encoded plasmid Rep proteins of the Rep_3 family (Table 1). Only the 11,127 bp pAC1839-2 from *A. baumannii* AC1839 harboured the *tetA(39)* tetracycline-resistance gene, while the other plasmids did not encode any AMR genes. An analysis of the pAC1839-2 structure is presented in Section 3.7.

### Overview of the large *bla*_NDM-1_-encoding plasmids in the four *A. baumannii* isolates

**Fig. 1.**
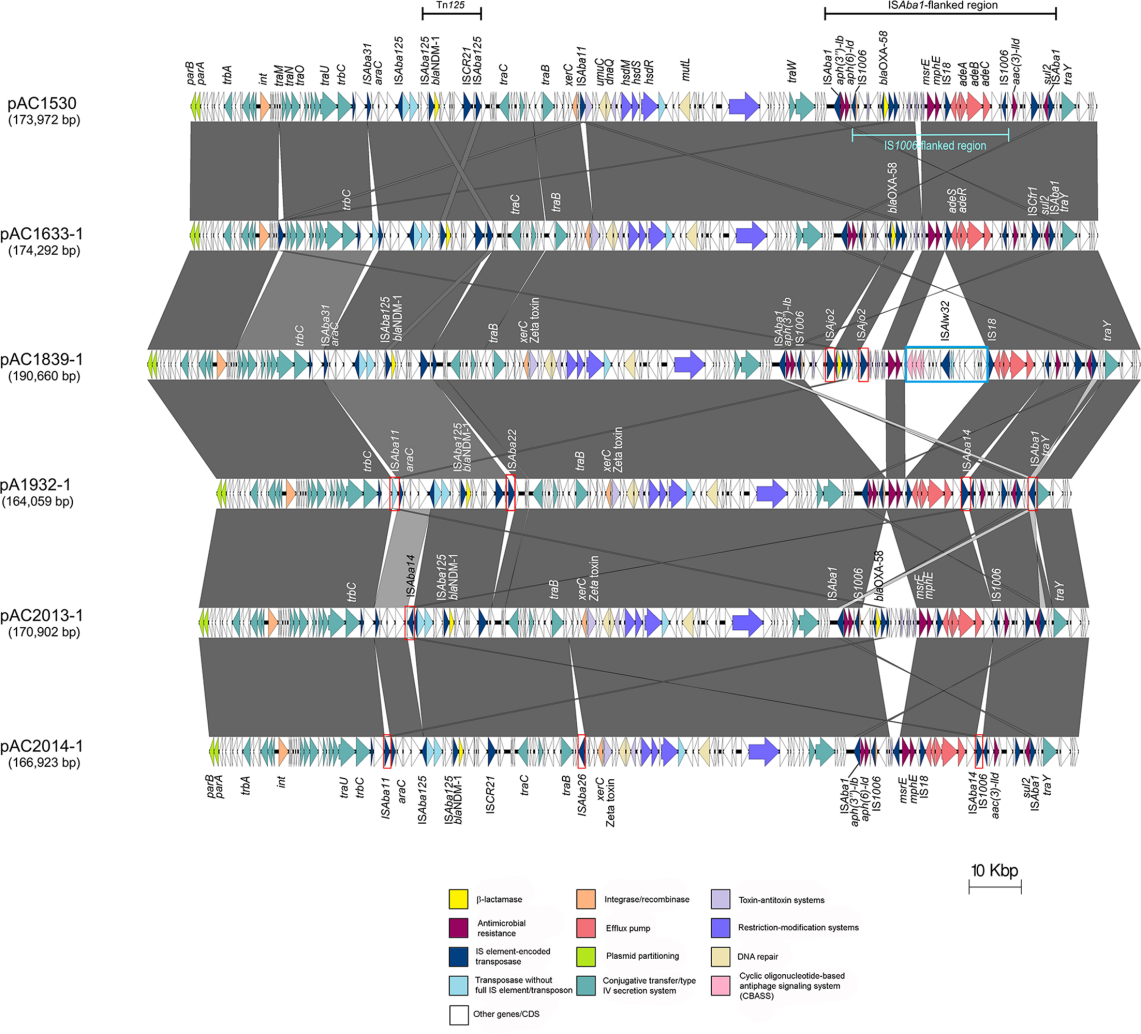
Comparative linear maps of the *bla*_NDM-1_-encoded plasmids identified in this study, along with plasmid pAC1530 from *A. nosocomialis* AC1530 (accession no. CP045561) and pAC1633-1 from *A. baumannii* AC1633 (accession no. CP059301), which were identified and presented previously [20]. Arrows represent genes/open reading frames that are coloured according to the legend provided. Grey-shaded areas between each linear plasmid map indicate regions that shared >97% nucleotide sequence identities. Red boxes indicate IS elements that were not shared among all plasmids, while the sky blue-coloured box outlined in the pAC1839-1 linear map referred to the ca. 15 kb putative transposon that contained IS*Alw32* and the type III CBASS phage defence system (see Section 3.5).

Comparison of the four large *bla*_NDM-1_-encoding plasmids identified in this study with two previously described *Acinetobacter* plasmids harbouring *bla*_NDM-1_ (i.e. pAC1530 and pAC1633-1) [20] revealed several structural differences (Fig. 1). Notably, a 29,670 bp region flanked by IS*1006* showed extensive variability. This region contains multiple *xrs* recombination sites and AMR genes, such as *bla*_OXA-58_ and *msrE–mphE*, and is inserted within a 14,750 bp composite transposon flanked by IS*Aba1*, designated Tn*6948*. Tn*6948* harbours additional AMR genes, including *sul2* and the aminoglycoside resistance genes *aac(3)-IId*, *aph(3'')-Ib* and *aph(6)-Id* [20]. The IS*1006* element was hypothesized to be the point of insertion (possibly through transposition or recombination) of a large segment from the *bla*_OXA-58_-encoding, 36 kb plasmid pOXA-58_AP882 (accession no. CP014479) into the 147 kb *bla*_NDM-1_-encoding plasmid pNDM-1_AP882 (accession no. CP014478). These plasmids were previously identified in an *A. pittii* clinical isolate from Perak, Malaysia, in 2014 [44], and were postulated to have given rise to the pAC1530/pAC1633-1 progenitor plasmid, which subsequently spread to *Acinetobacte*r isolates in Terengganu [20].

Among the plasmids harbouring *bla*_NDM-1_, pAC1839-1 was the largest, at 190,660 bp. A distinctive feature of this plasmid is the insertion of a unique 15,434 bp fragment between the *bin* resolvase gene and IS*18* within the IS*1006*-flanked region. This fragment appears to represent a novel transposon-like element exclusive to pAC1839-1 and is described in detail in Section 3.5. Additionally, pAC1839-1 harbours two copies of IS*Ajo2* near the *bla*_OXA-58_ gene, which are absent in the other plasmids (Fig. 1). In the remaining three *bla*_NDM-1_-encoding plasmids, most of the structural variations were also confined to the IS*1006*-flanked region, which is rich in *xrs* recombination sites. Notably, the *bla*_OXA-58_ gene and several surrounding genes in *xrs* modules are absent in both pAC1932-1 and pAC2014-1 (Fig. 1). Further details regarding the insertions and deletions involving *xrs* modules are presented in Section 3.6.

Besides the variations within the IS*1006*-flanked *xrs*-rich regions, other structural changes include the insertions of various IS elements. Both pAC1932-1 and pAC2014-1 have the 1,282 bp IS*Aba14* inserted upstream of the IS*1006* copy, which is located one gene away from the *aac(3)-IId* aminoglycoside resistance gene (Fig. 1). Both plasmids also have the 1,101 bp IS*Aba11* inserted adjacent to the IS*Aba31* found upstream of the *araC* gene; in pAC1633-1, there is a partial IS element with an IS*4* family transposase inserted near the same location, and this insertion led to the truncation of IS*Aba31* (Fig. 1) [20]. An IS*Aba1* is found upstream of the *traY* gene in pAC1932-1, but this is not seen in pAC2014-1 or pAC2013-1. On the other hand, pAC2014-1 carries the insertion of IS*Aba26* (1,318 bp, an element of the IS*256* family) near the *traB* gene, while pAC2013-1 contains IS*Aba14* (1,282 bp; IS*3* family) next to IS*Aba125* upstream of the Tn*125* transposon, which carries the *bla*_NDM-1_ gene (Fig. 1). IS*Aba26* and IS*Aba14* are absent in the other *bla*_NDM-1_-encoded plasmids.

A SNP analysis was conducted on the core regions of the six *bla*_NDM-1_-encoded plasmids, with pAC1530 as the reference plasmid, revealing low SNP counts of between 2 and 200 (Table S2), thus inferring that the plasmids are highly conserved. Interestingly, pAC1633-1 had only two SNP differences compared with pAC1530, indicating that these two plasmids are nearly identical and underlining the suggestion that the plasmid was horizontally transmitted between *A. nosocomialis* AC1530 and *A. baumannii* AC1633 [20]. A phylogenetic tree generated from the pairwise core SNP differences of the plasmids (Fig. S3) supported the close relatedness of the six plasmids, which shared a recent common ancestor. pAC1932-1 (without *bla*_OXA-58_) was the most divergent, with 200 SNP differences when compared with pAC1530, and between 121 and 129 SNP differences when compared with the other three plasmids (including the other plasmid that was absent for *bla*_OXA-58_, pAC2014-1). Plasmid copy numbers were estimated by mapping the respective plasmid short reads to the assembled complete genomes as the reference and analysing the coverage depth relative to the chromosome, leading to average values of between 1.4 and 1.7 (Table S3).

### The *bla*_NDM-1_ gene is found in variant Tn*125* structures

**Fig. 2.**
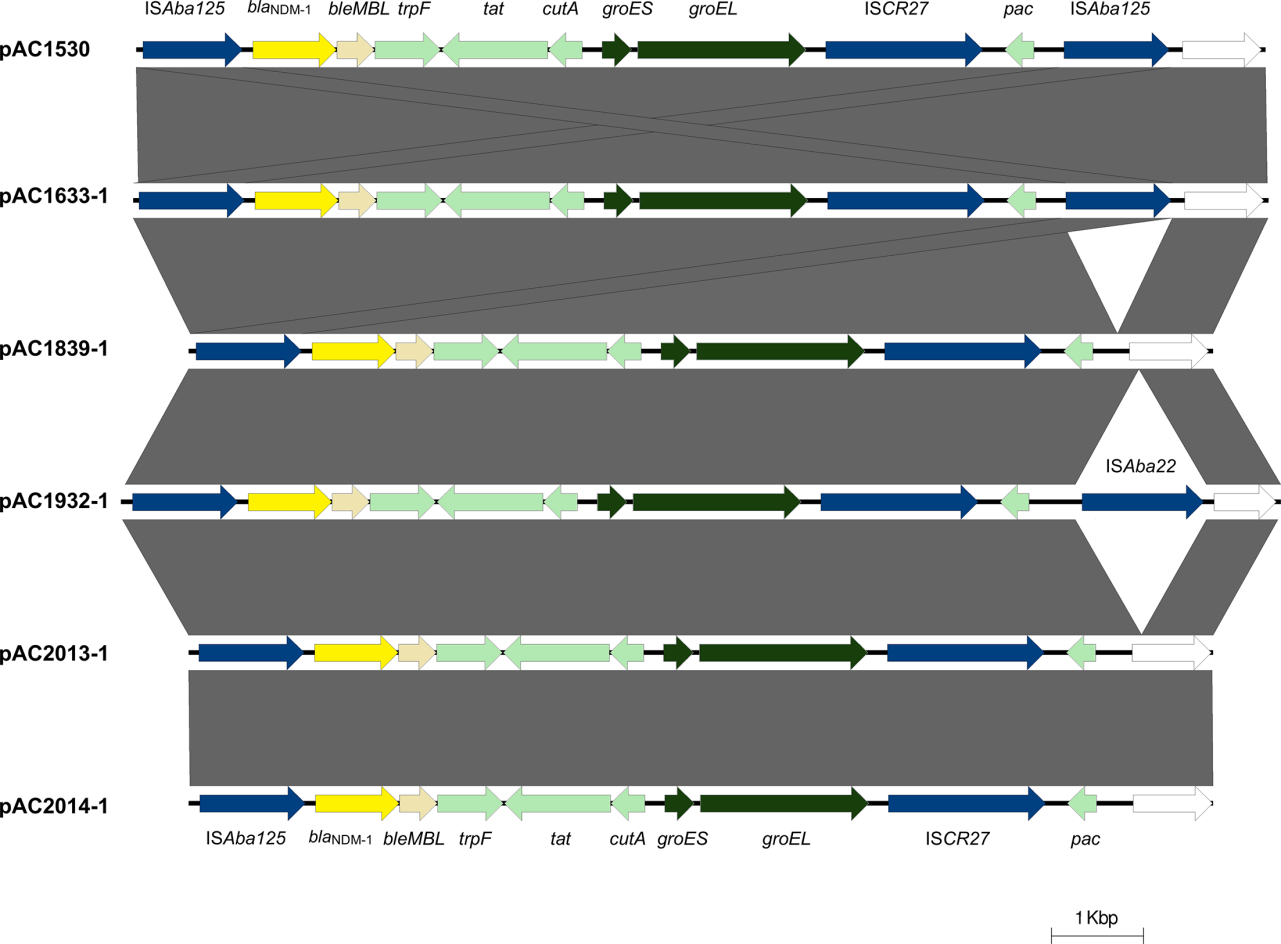
Comparative linear map of the Tn*125* structure found in the *bla*_NDM-1_-encoded plasmids pAC1530 and pAC1633-1 identified in the genomes of *A. nosocomialis* AC1530 and *A. baumannii* AC1633, respectively [20]), and in the plasmids identified from *A. baumannii* AC1839, AC1932, AC2013 and AC2014 in this study. Grey-shaded areas between the linear plasmid maps indicate regions of 100% nucleotide sequence identities. IS element-encoded transposases are indicated in dark blue arrows; the *bla*_NDM-1_ gene is depicted as a yellow arrow, with its downstream *ble*_*MBL*_ bleomycin-resistance gene as a beige arrow. Genes encoding the GroES and GroEL chaperonin proteins are shown in dark green arrows, while light green arrows indicate genes with known functions, and white arrows indicate reading frames encoding hypothetical proteins.

The most common vehicle for the dissemination of the *bla*_NDM-1_ carbapenemase gene in *Acinetobacter* spp. is the composite transposon Tn*125*, which is usually flanked by IS*Aba125* [14,18, 45] and was found in both pAC1530 and pAC1633-1 [20]. However, in the four NDM-1-positive *A. baumannii* isolates, Tn*125* had lost the IS*Aba125* located downstream of *bla*_NDM-1_. In pAC1932-1, this copy of IS*Aba125* was replaced by IS*Aba22* (Fig. 2). Variants of Tn*125* have been previously reported with the loss of one copy of IS*Aba125* in a ca. 46 kb plasmid pNDM-BJ02 from a clinical isolate of *Acinetobacter lwoffii* from China [46] and in a ca. 64 kb plasmid in *A. baumannii* DT01139, isolated from a blood culture of a neonate in Tanzania [47]. In the case of the pAP-D499 plasmid of an *A. pittii* isolate from China, the downstream IS*Aba125* element was replaced by IS*Aba11* [48], whereas in *A. baumannii* AB-NDM-1, isolated in Spain, Tn*125* was located on the chromosome and was flanked by IS*Aba125* and IS*Aba14* [49].

### A novel 15 kb transposon-like element with a phage defence system was inserted into pAC1839-1

**Fig. 3.**
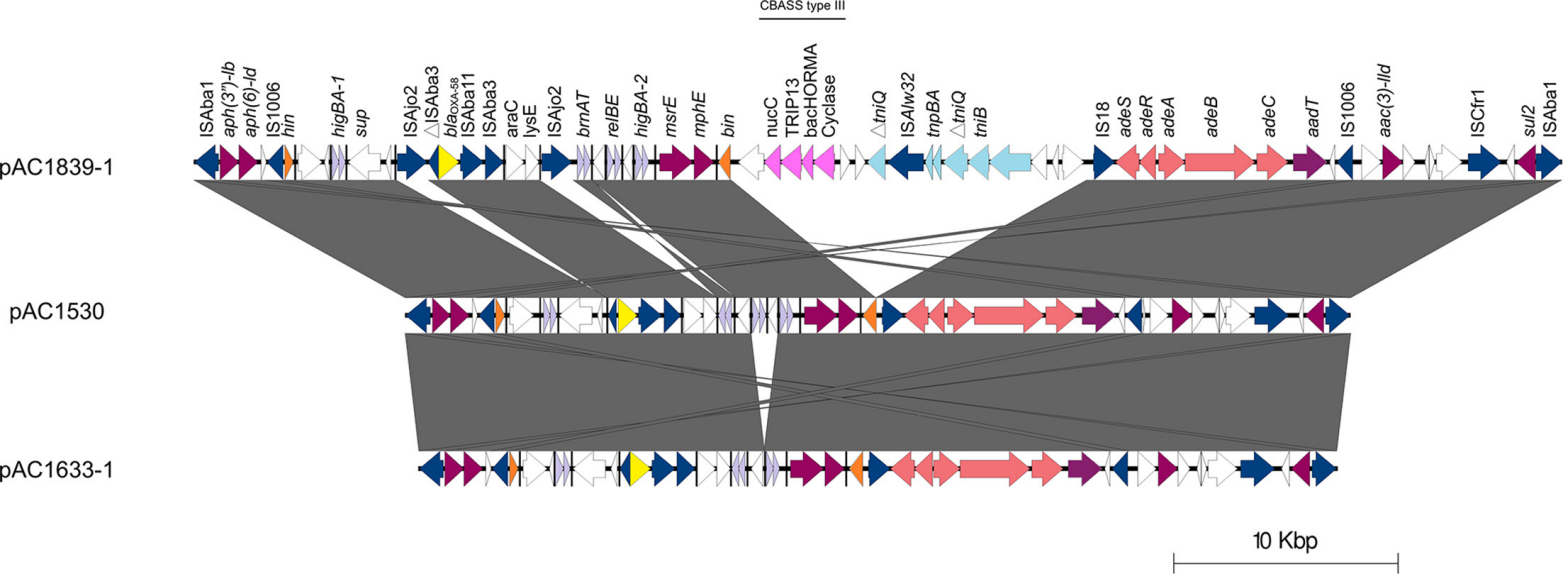
Comparative linear genetic map of the IS*Aba1*-flanked regions of the *bla*_NDM-1_-encoded plasmids pAC1839-1 with pAC1530 and pAC1633-1, showing the putative 15,434 kb transposon inserted between the *bin* recombinase and IS*18* in pAC1839-1. AMR genes are indicated in maroon-coloured arrows, except *bla*_OXA-58_, which is depicted as a yellow arrow. Efflux-mediated genes are shown as salmon-coloured arrows; IS-encoded transposases are indicated as dark blue arrows, while the transposases found within the 15,434 bp transposon are shown as light blue arrows. Toxin-antitoxin genes are shown as light purple arrows, while genes that made up the type III CBASS system are depicted as pink arrows and labelled. White arrows represent hypothetical genes. Vertical bars on the linear maps indicate *xrs* sites. The extent of regions with >99% nucleotide sequence identities is indicated in the grey-shaded area between each linear map.

In pAC1839-1, there is an additional 15,434 bp fragment between the *bin* resolvase and IS*18* within an *xrs*-rich 29,670 bp region flanked by IS*1006* (Fig. 3). Numerous transposases and transposase-like sequences were found within this 15.4 kb region. Three transposase genes identified by ISfinder belong to a 2,512 bp IS*Alw32* element of the IS*66* family, which is flanked by 16 bp inverted repeats. IS*Alw32* was first described in *A. lwoffii* M2a, isolated from honey [50]. In pAC1839-1, IS*Alw32* was inserted into a *tniQ*-encoded putative transposase, and this insertion led to an 8 bp direct repeat of the target site (5′-ATTTCTTT-3′). An intriguing set of four genes belonging to the cyclic oligonucleotide-based antiphage signalling system (CBASS) was identified within this 15.4 kb, transposase-rich region in pAC1839-1 (Fig. 3). CBASS is a phage defence system in bacteria and archaea that utilizes cGAS/DncV-like nucleotidyltransferase (CD-NTase, also known as cyclase) proteins to sense phage infection. It is activated to synthesize a cyclic nucleotide immune signal, which then binds to Cap (CD-NTase-associated protein) effectors that induce host cell death to inhibit phage propagation, as reviewed in [51]. CBASS systems are highly diverse, with four types that have been described so far; all four types have the CD-NTase and Cap effector genes as their core, with genes encoding different additional Cap proteins that regulate the CD-NTase function as ancillary proteins [51,52]. The CBASS system found in pAC1839-1 belongs to type III, as it includes ancillary genes that encode Cap proteins with HORMA and TRIP13 domains [52], in addition to the core CD-NTase and Cap effector genes (Fig. 3). The pAC1839-1-encoded type III CBASS system is not found in the other four *A. baumannii* genomes analysed in this study (i.e. AC1633, AC1932, AC2013 and AC2014). Nevertheless, a blastn search of the other 122 *A. baumannii* genomes from the same hospital, as published in [26], showed a hit with *A. baumannii* AC2010 (contig_39; accession no. JAQIPT0000000039.1) and only for the four genes that made up the type III CBASS (at 80% sequence similarity). The IS*Alw32* sequence was identified across three contigs (contig_70, contig_77 and contig_80), and no other sequences from the 15.4 kb region of pAC1839-1 could be found in the AC2010 genome. We are also unable to ascertain whether the type III CBASS system in AC2010 is plasmid or chromosomally encoded due to the low contiguity of the short-read assembly. This suggests the rarity of the type III CBASS system in our *A. baumannii* collection, and in AC1839, it was likely acquired along with the transposon-like element that had inserted into pAC1839-1. Unfortunately, very little is known about CBASS and most other phage defence systems in *Acinetobacter* spp. [53]. Although a very comprehensive *in silico* survey of phage defence systems and their hotspots in *Acinetobacter* spp. was recently published [54], their functional characterization awaits further investigations.

### Rearrangements surrounding the *bla*_OXA-58_ region likely driven by Xer recombination

In addition to the *bla*_NDM-1_ carbapenemase gene, plasmids pAC1530 and pAC1633-1 also co-harboured the *bla*_OXA-58_ class D carbapenemase gene, and a hallmark of this gene is its location within a *xrs* module flanked by XerC–XerD recombination sites [20]. The region surrounding *bla*_OXA-58_ is rich in XerC–XerD sites (or *xrs* sites) Table 3 with *xrs* modules harbouring toxin–antitoxin systems, the *msrE*–*mphE* macrolide resistance genes, and others found within a 29 kb region surrounding *bla*_OXA-58_ that is flanked by IS*1006* [20]. When the plasmids from this study were compared with pAC1530 and pAC1633-1, several rearrangements were observed within this region.

**Fig. 4.**
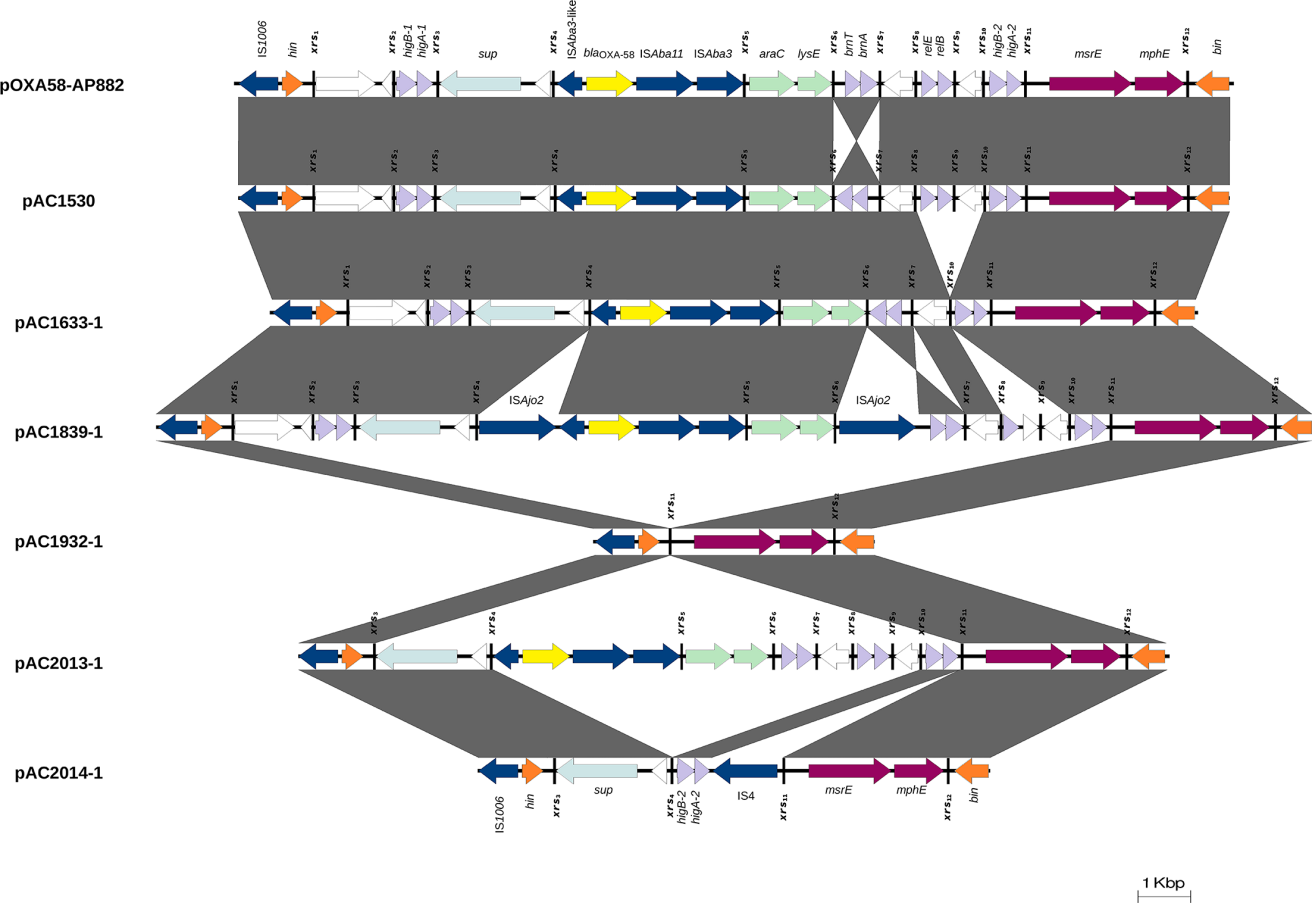
Comparative linear map of the *xrs*-rich regions surrounding *bla*_oxa-58_ in pAC1839-1 and pAC2013-1, with reference plasmids pAC1530 from *A. nosocomialis* AC1530, pAC1633-1 from *A. baumannii* AC1633 [20] and pOXA58-AP882 from *A. pittii* AP882 [44]. AMR genes are depicted as maroon arrows, except *bla*_OXA-58_, which is shown as a yellow arrow. IS element-encoded transposases are shown as dark blue arrows and labelled accordingly. Light purple-coloured arrows depict toxin–antitoxin systems, while orange arrows indicate putative recombinases. Light green and light blue arrows indicate genes with known functions, and white arrows indicate reading frames encoding hypothetical proteins. *xrs* sites are shown as vertical bars and labelled as listed in Table S4, which details the respective *xrs* nucleotide sequences.

Only two of the four plasmids characterized in this study (namely pAC1839-1 and pAC2013-1) were found to harbour both the *bla*_NDM-1_ and *bla*_OXA-58_ genes; in the other two plasmids (i.e. pAC1932-1 and pAC2014-1), the *bla*_OXA-58_ gene was absent. The *xrs* sites are numbered here according to their order in pAC1530, which had two additional *xrs* sites compared with pAC1633-1 [20]. In pAC1932-1, there is an absence of an 11.5 kb fragment spanning the *xrs*_1_ site downstream of the *hin* recombinase to the *higBA-2* toxin–antitoxin genes upstream of the *xrs*_11_ site, leaving only the *msrE–mphE* macrolide resistance module flanked by *xrs*_11_ and *xrs*_12_ (Fig. 4). A smaller deletion was observed in pAC2014-1, which involved a 7.7 kb fragment spanning the *xrs*_4_ site downstream of the *sup* gene to the hypothetical gene that was flanked by the *xrs*_9_ and *xrs*_10_ sites in pAC1530 (Fig. 4). In addition, there was an insertion of IS*4* immediately downstream of the *higBA-2* toxin–antitoxin genes located in between the *xrs*_4_ and *xrs*_11_ sites of pAC2014-1 (Fig. 4). The absence of *bla*_OXA-58_ in *A. baumannii* AC1932 and AC2014 does not appear to affect their resistance to carbapenems, as both isolates showed similar MIC levels for meropenem (>32 µg ml^−1^), imipenem (>8 µg ml^−1^) and ertapenem (>1 µg ml^−1^) compared with their *bla*_OXA-58_-positive counterparts (i.e. AC1633, AC1839 and AC2013) (Table 2). This suggests that in these *A. baumannii* isolates, carbapenem resistance was primarily attributed to the *bla*_NDM-1_ gene*.*

When comparing pAC2013-1 with pAC1530, a 2.2 kb fragment that spans the *xrs*_1_ to *xrs*_3_ sites and covers the *higBA-1* toxin–antitoxin genes was absent. However, the remaining regions that span *sup*, *bla*_OXA-58_, until the *higBA-2* and *msrE–mphE* genes remain intact (Fig. 4). In the case of pAC1839-1, insertions of two copies of the 1,482 bp IS*Ajo2* were observed: one copy is located immediately after the *xrs*_4_ site, upstream of the partial IS*Aba3* element found next to *bla*_OXA-58_. This insertion of IS*Ajo2* led to a 6 bp AGAGAG duplication of the insertion site. Another copy of IS*Ajo2* is found after the *xrs*_6_ site (its insertion resulting in a 5 bp GCAGC target site duplication) and is located upstream of the *brnTA* toxin–antitoxin *xrs* module, which is in inverted orientation compared with its counterparts in pAC1530 and pAC1633-1 (Fig. 4). One of the differences between pAC1530 and pAC1633-1 is the absence of the *relBE* toxin–antitoxin genes, which were flanked by the *xrs*_8_ and *xrs*_9_ sites, and a hypothetical gene flanked by *xrs*_9_ and *xrs*_10_ in pAC1633-1 [20]; these genes are, however, present in pAC1839-1 as well as pAC2013-1 (Fig. 4). The organization and orientation of the *xrs* sites are almost identical in all the plasmids analysed; comparative analysis of the 28 bp core regions of the *xrs* sites showed only up to two mismatched nucleotides within the XerC and/or XerD binding sites (highlighted in yellow in the sequences shown in **Table S4**).

### The *tetA(39)* tetracycline resistance gene in pAC1839-2 is also located on a mobile *xrs* module

**Fig. 5.**
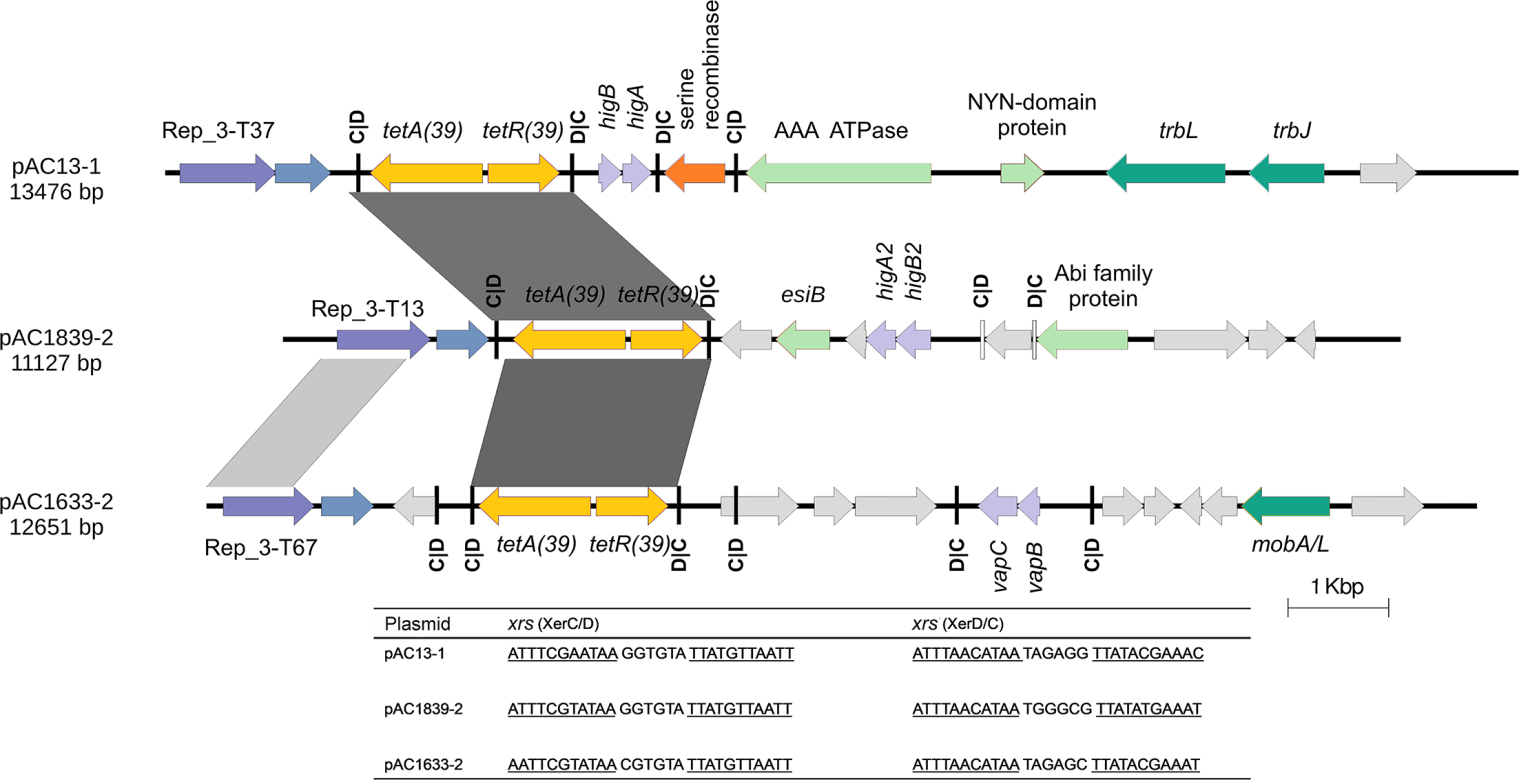
Linear maps of the plasmids harbouring the *tetA(39)-tetR(39*) *xrs* module from *Acinetobacter* spp. clinical isolates from HSNZ. The plasmids shown are pAC13-1 from *A. nosocomialis* AC13, which was isolated in 2011 [55]; pAC1633-2 from *A. baumannii* AC1633, isolated in 2016 [20] and pAC1839-2 from *A. baumannii* AC1839, isolated in 2018 (this study). The plasmid replicase gene is indicated in purple arrows, while its downstream gene, designated *orfX*, is shown in dark blue arrows. The *tetA(39)-tetR(39)* tetracycline resistance genes are shown as yellow arrows. Toxin–antitoxin genes are depicted in light purple arrows; plasmid mobilization-related genes are shown in green arrows; and light green arrows indicate genes with known functions or domains. NYN-domains are protein domains typified by the eukaryotic Nedd4-binding protein 1 and the bacterial YacP-like proteins (Nedd4-BP1, YacP nucleases). Orange arrows depict putative recombinases. The *xrs* sites are indicated as thick vertical lines, labelled as C|D for XerC–XerD and D|C for XerD–XerC recognition sites. The nucleotide sequence for the *xrs* sites flanking the *tetA(39)-tetR(39)* genes is shown below the linear map.

*A. baumannii* AC1839 also harboured the *tetA(39)*-*tetR(39)* gene pair in a smaller plasmid designated pAC1839-2 (11,127 bp), which is classified as an R3-T13-type plasmid based on the *Acinetobacter* plasmid classification scheme [42]. Like the *bla*_OXA-58_ gene in pAC1839-1, the *tetA(39)*-*tetR(39)* gene pair is located within a *xrs* module (Fig. 5). The 2,001 bp *tetA(39)*-*tetR(39) xrs* module has been found in several *Acinetobacter* plasmids [24], and we have previously reported its presence in a 13,476 bp plasmid of the R3-T37 group designated pAC13-1 in three *A. nosocomialis* isolates from HSNZ in 2011 [55] and a 12,651 bp plasmid of the R3-T67 group designated pAC1633-2 in *A. baumannii* AC1633, isolated from the same hospital in 2016 [20]. Comparison of these three plasmids showed sequence similarity only in the *tetA(39)*-*tetR(39)* genes within the *xrs* module (Fig. 5), inferring the mobility of the tetracycline resistance *xrs* module among different strains of *Acinetobacter* spp. in HSNZ since 2011. The *xrs* sequences flanking the *tetA(39)*-*tetR(39)* genes were also highly similar (Fig. 5). The *tetA(39)* gene conferred resistance to tetracycline but not to doxycycline or minocycline [24,55], and this was also observed in *A. baumannii* AC1839 (Table 2) [26].

## Discussion

Ever since the first report of NDM-1 in *Klebsiella pneumoniae* 05–506, isolated in New Delhi, India, in 2008 [56], NDM-1 and its many variants have been reported in various species and have spread worldwide [57]. *A. baumannii* clinical isolates harbouring the *bla*_NDM-1_ were first reported in India [58] and China [59] in 2010. Although OXA-23 is the prevalent acquired carbapenemase in clinical *A. baumannii* isolates in most parts of the world, there have been increasing reports of *A. baumannii* producing NDM-1 in recent years. In these cases, the *bla*_NDM-1_ gene is often found within Tn*125* on large, transmissible plasmids that serve as vehicles for its dissemination [8,15, 60]. We previously reported a ca. 170 kb plasmid harbouring *bla*_NDM-1_ along with *bla*_OXA-58_ and several other AMR genes in *A. nosocomialis* AC1530 and *A. baumannii* AC1633 from HSNZ in Terengganu, Malaysia [20]. Subsequent genome sequencing of other *A. baumannii* isolates from the same hospital revealed four additional *A. baumannii* isolates that carry *bla*_NDM-1_. However, the data were obtained from short-read assemblies [26], which made it challenging to piece together the complete genetic structure of the plasmid(s) that harboured the gene. In this study, hybrid assembly with long-read Nanopore sequencing data enabled us to resolve the complete structure of these plasmids, allowing the dynamics of the *bla*_NDM-1_-encoded plasmid to be investigated as it was transmitted within the same hospital between 2015 and 2020.

Several large, conjugative *Acinetobacter* plasmids carrying *bla*_NDM-1_ have been characterized, and recent comparative studies have highlighted their remarkable diversity [8,15]. Tang *et al*. [8] identified a 45,911 bp plasmid in *A. junnii* YR7, designated pNDM-YR7, which harbours *bla*_NDM-1_ within a truncated Tn*125* that lacked the downstream IS*Aba125*. They also identified 30 additional *Acinetobacter* plasmids that shared a similar conjugative transfer region and origin of transfer (*oriT*) with pNDM-YR7. Notably, none of these plasmids contained an identifiable *rep* gene, an observation similar to the *bla*_NDM-1_ plasmids described in this study. In addition, Tang *et al*. [8] identified 32 other *bla*_NDM-1_-harbouring plasmids that differ from pNDM-YR7 in various *Acinetobacter* spp. Among these, 19 of which encoded a replicase gene of the Rep_3 family. Rodrigues *et al*. [15] described a very large, ca. 340 kb plasmid, pCCBH31258, which harboured *bla*_NDM-1_ within a complete Tn*125* structure, as well as *bla*_OXA-58_ embedded in an *xrs* module that differs from the module found in the Malaysian plasmids. In pCCBH31258, the *bla*_OXA-58_ *xrs* module comprises IS*Aba3*-*bla*_OXA-58_-ΔIS*Aba3*-IS*Api2* [15], whereas in pAC1839-1 and the other Malaysian NDM-1 plasmids, the *xrs* structure comprises ΔIS*Aba3*-*bla*_OXA-58_-IS*Aba11*-IS*Aba3*. Rodrigues *et al*. [15] also identified a *rep* gene in pCCBH31258, which reportedly does not conform to the current *Acinetobacter* plasmid classification scheme [42,43]. Four other *A. baumannii* isolates from the same hospital in Brazil harbour plasmids similar to pCCBH31258, but their complete structure could not be resolved due to fragmented assembly from Illumina short-read data [15]. Intriguingly, five other NDM-1-producing *A. baumannii* isolates sequenced in the same study were found to possess plasmids with a distinct genetic background from pCCBH31258 and other known *Acinetobacter* NDM-1 plasmids [15]. These findings underscored the considerable genetic diversity of plasmids that harbour the *bla*_NDM-1_ gene in *Acinetobacter* spp.

The comparative analysis of the large *bla*_NDM-1_-encoded plasmids in this study highlights their structural fluidity, mediated by various mobile elements, as they move between different strains. Notably, all five *A. baumannii* isolates carrying this plasmid were clonally unrelated, strongly suggesting horizontal transfer of the plasmid, which was initially identified in *A. nosocomialis* AC1530. Genes associated with conjugative transfer were identified in plasmids pAC1530 and pAC1633-1, sharing 50–70% amino acid sequence identity with the corresponding conjugative transfer proteins of plasmid pA297-3 from *A. baumannii* A297 [20,61]. The same conjugative transfer genes were also found in the four large plasmids analysed in this study. Although functional validation through plasmid transfer experiments (e.g. conjugation or transformation into a carbapenem-susceptible *A. baumannii* strain) could further validate the resistance phenotype, such assays had previously been performed and reported [16]. In our earlier work, conventional conjugation assays using azide-resistant, carbapenem-susceptible *A. baumannii* recipients did not yield transconjugants for plasmids pAC1633-1 and pAC1530, despite multiple attempts [20]. This finding was attributed to the insertion of a 42 kb fragment containing the IS*Aba1*-flanked Tn*6948* element, which disrupted the conjugative transfer region and likely reduced the transfer efficiency below detectable levels. Given these prior findings and the current study’s focus on comparative genomic characterization rather than functional assays, additional plasmid transfer experiments were not repeated here. Nevertheless, the detection of similar plasmids among clonally distinct *A. baumannii* isolates over subsequent years provides strong genomic evidence of horizontal dissemination within the hospital environment.

One of the structural variations discovered in these *bla*_NDM-1_-encoded plasmids lies within the Tn*125* transposon that harbours *bla*_NDM-1_. In most *Acinetobacter* isolates, including *A. nosocomialis* AC1530 and *A. baumannii* AC1633, Tn*125* is flanked by two IS*Aba125* elements. However, in three of the four newly characterized *bla*_NDM-1_-encoding plasmids, only a single IS*Aba125* remains. In the fourth plasmid, pAC1932-1, one of the IS*Aba125* elements has been replaced by IS*Aba22*. In all four plasmids, the IS*Aba125* that remained was the copy located immediately upstream of *bla*_NDM-1_ (Fig. 2). This copy of IS*Aba125* provides the external promoter that drives the expression of *bla*_NDM-1_ [9], which accounts for the carbapenem-resistant phenotype observed in all four *A. baumannii* isolates. The production of MBLs in these isolates was confirmed using the Etest MBL kit (bioMérieux, La Balme-les-Grottes, France). Such structural variations in Tn*125* have been reported previously, and in these reports, it was always the IS*Aba125* downstream of *bla*_NDM-1_ that was either absent or replaced by another IS element [8,46, 48, 49]. The IS*Aba125* upstream of *bla*_NDM-1_ is required for its expression and is, thus, more often conserved than other genes or elements within Tn*125* [45].

Two of the *A. baumannii* HSNZ isolates (i.e. AC1839 and AC2013) also co-harboured the *bla*_OXA-58_ carbapenemase gene, similar to *A. nosocomialis* AC1530 and *A. baumannii* AC1633 [20]. In contrast, *bla*_OXA-58_ was absent in the genomes of AC1932 and AC2014. Despite this variation, all four *A. baumannii* isolates, along with *A. baumannii* AC1633, exhibit carbapenem resistance with comparable MIC values for imipenem, meropenem and ertapenem (Table 2), indicating that *bla*_NDM-1_ is likely the primary driver of carbapenem resistance in these isolates. In *A. pittii* 44551 that co-harbours *bla*_NDM-1_ and *bla*_OXA-58_ in separate plasmids, carbapenem resistance was attributed to *bla*_NDM-1_, while *bla*_OXA-58_ remained silent, likely due to the absence of an upstream promoter to drive its expression in plasmid pOXA58-44551 [62]. In the *A. pittii* 44551 plasmid pNDM-44551, *bla*_NDM-1_ was found within a truncated Tn*125* structure, whereby the IS*Aba125* downstream of *bla*_NDM-1_ was absent, but the IS*Aba125* upstream of *bla*_NDM-1_, which provided the promoter for its expression, was present [62]. The sequences immediately upstream of *bla*_OXA-58_ in pAC1839-1, pAC2013-1 and pAC1633-1 were identical to pOXA58-44551 (i.e. a 369 bp truncated IS*Aba3* adjacent to *bla*_OXA-58_). This suggests that *bla*_OXA-58_ may be transcriptionally silent or expressed at very low levels in pAC1839-1, pAC2013-1 and pAC1633-1, but such a postulation warrants further experimental validation through transcriptomic analysis, which is beyond the scope of this paper. Nonetheless, the genomic data, together with biochemical confirmation of MBL activity (via the Etest MBL kit and the BD Phoenix M50 susceptibility test results), provide strong evidence supporting the functional relevance of the identified carbapenemase genes.

Downstream of *bla*_OXA-58_, the genetic environment differs. In the Malaysian *A. baumannii* plasmids, *bla*_OXA-58_ is followed by IS*Aba11* and IS*Aba3*, forming the *bla*_OXA-58_ *xrs* module (see Fig. 4), whereas IS*Aba11* is absent in pOXA58-44551, and its *xrs* module comprises only of ΔIS*Aba3*-*bla*_OXA-53_-IS*Aba3* [62]. Although *xrs* modules have been found in various bacterial genera, they are most extensively characterized in *Acinetobacter*, where their frequent transmission has been well documented [21,39]. Alongside IS elements, integrons and transposons, *xrs* modules contribute significantly to the evolutionary dynamics of *Acinetobacter* plasmids [39,63]. These *xrs* modules are the target sites for the XerC–XerD site-specific tyrosine recombinase system. While the canonical function of this system is to resolve plasmid multimers into monomers to ensure stable inheritance [64], it is increasingly recognized as a key driver of plasmid structural evolution. The presence of compatible *xrs* sites on different plasmids allows the system to be hijacked, mediating inter-molecular recombination and fostering the formation of plasmid cointegrates. The subsequent resolution of these cointegrates can then facilitate the exchange and shuffling of genetic modules bounded by *xrs* sites [25,65]. As recently highlighted by Blanchais *et al.* [22], this interplay between the Xer system and plasmid-carried *xrs* sites is a powerful mechanism for the dissemination of antibiotic resistance. Moreover, the presence of a plasmid-encoded *xerC* gene, as we identified in the large *bla*_NDM-1_ plasmids of this study (Fig. 1), likely enhances this process. It provides the necessary recombinase encoded by the plasmid itself, making the formation of cointegrates and subsequent gene exchange less dependent on the host chromosomal Xer machinery. Our findings support this, as the movement of the *bla*_NDM-1_-encoded large plasmid across different *A. baumannii* clonal lineages within the same hospital showcases structural fluidity mostly driven by the combined activity of IS elements and Xer-mediated recombination at *xrs* modules. Further work will be required to validate this hypothesis experimentally. For instance, creating a targeted knockout of the plasmid-encoded *xerC* gene and assessing its impact on plasmid cointegrate formation and *xrs* module mobility in a suitable *Acinetobacter* host would provide direct evidence of its specific function.

In conclusion, this study builds upon our previous work that characterized a large, ca. 170 kb plasmid encoding *bla*_NDM-1_, *bla*_OXA-58_ and a host of other AMR genes, originally identified in carbapenem-resistant, MDR *A. nosocomialis* and *A. baumannii* in HSNZ in 2015 and 2016, respectively [20]. Genome sequencing of *A. baumannii* isolates from the same hospital in subsequent years revealed that this plasmid continues to be transmitted, as it is now found in distinct, clonally unrelated *A. baumannii* lineages. Comparative analysis of the complete plasmid sequences demonstrates structural fluidity, primarily mediated by IS elements and *xrs* modules. In two of these plasmids (pAC1932-1 and pAC2014-1), the *bla*_OXA-58_ *xrs* module and several adjacent *xrs* modules are absent. However, there were no differences in carbapenem MIC values between these isolates and those harbouring both *bla*_NDM-1_ and *bla*_OXA-58_, suggesting that *bla*_NDM-1_ is the primary contributor to the carbapenem resistance phenotype. The *bla*_OXA-58_ gene is likely either silent or expressed at very low levels, possibly due to the absence of an upstream promoter. Transcriptomic analysis would provide clarity on the expression dynamics of these carbapenemase genes, and the absence of such data represents a limitation of this study. Thus, transcriptomic profiling of these *A. baumannii* strains is recommended as an important direction for future research.

In plasmid pAC1839-1, a novel presumptive 15 kb transposon that harbours a type III CBASS phage defence system was identified, although its function and biological significance remain currently unknown. *A. baumannii* AC1839 also harbours an additional plasmid, pAC1839-2, which carries the *tetA(39)-tetR* tetracycline resistance gene pair in a *xrs* module. This identical module has been found in several *Acinetobacter* isolates within diverse Rep_3 family plasmids, underscoring its mobility. What we proposed 4 years ago in our initial report [20], that this plasmid is capable of ongoing horizontal transfer, remains valid today. Thus far, the plasmid has only been identified in non-Global Clone (non-GC) *A. baumannii* lineages. However, in HSNZ and many hospitals globally, the predominant lineage is Global Clone 2 (GC2) [26,60]. Should this plasmid be acquired by a GC2 strain, its dissemination potential would likely increase significantly. These findings underscore the urgent need for continuous genomic surveillance, particularly for a pathogen that remains at the top of the World Health Organization’s critical priority pathogen list [3], and also highlight the utility of long-read sequencing in understanding the complex genomic rearrangements that underlie the lateral transfer of AMR genes and other mobile elements.

## Supplementary material

10.1099/mgen.0.001630 Uncited Supplementary Material 1.

## Abbreviations

AMR: antimicrobial resistance
CBASS: cyclic oligonucleotide-based antiphage signalling system
CD-NTase: cGAS/DncV-like nucleotidyltransferase
CPOs: carbapenemase-producing organisms
CRAB: carbapenem-resistant *Acinetobacter baumannii*
DNBSeq: DNA nanoball sequencing
EARS-Net: European antimicrobial resistance surveillance network
GC2: global clone 2
GFF: general feature format
HSNZ: hospital sultanah nur zahirah
IS: insertion sequence
MBL: metallo-β-lactamase
MDR: multidrug resistant
MIC: minimum inhibitory concentration
MLST: multilocus sequence typing
NCBI: national center for biotechnology information
NDM: New Delhi metallo-β-lactamase
non-GC: non-global clone
OXAs: oxacillinases
xrs: xer recombination site
