# Supplementary material for: Dynamics of a large multidrug-resistant plasmid encoding New Delhi metallo-β-lactamase-1 and oxacillinase-58 carbapenemases in Acinetobacter baumannii clinical isolates from a tertiary hospital in Malaysia
Source: Microb Genom. 2026 Feb 11;12(2):001630. doi: 10.1099/mgen.0.001630 (PMC12893667; doi:10.1099/mgen.0.001630)
Supplement: Uncited Supplementary Material 1. [file mgen-12-01630-s001.pdf]

Supplementary Data

Dynamics of the large multidrug-resistant plasmid encoding the NDM-1 and OXA-58 carbapenemases in *Acinetobacter baumannii* clinical isolates from a tertiary hospital in Malaysia

Nurul Saidah Din[1], Farahiyah Mohd Rani[1], Salwani Ismail[1], Nor Iza A. Rahman[1], Hong Leong Cheah[2,3], Hock Siew Tan[2,3], Sadequr Rahman[2,3], David W. Cleary[4,5], Qasim Ayub[2,3], Stuart C. Clarke[1,6,7,8,9]* and Chew Chieng Yeo[1]*

[1]Centre for Research in Infectious Diseases and Biotechnology (CeRIDB), Faculty of Medicine, Universiti Sultan Zainal Abidin, Kuala Terengganu, Malaysia; [2]School of Science, Monash University Malaysia, Bandar Sunway, Malaysia; [3]Monash University Malaysia Genomics Platform, Bandar Sunway, Malaysia; [4]Department of Microbes, Infections and Microbiomes, School of Infection, Inflammation and Immunology, College of Medicine and Health, University of Birmingham, Birmingham, United Kingdom; [5]Institute of Microbiology and Infection, University of Birmingham, Birmingham, United Kingdom; [6]Faculty of Medicine and Institute for Life Sciences, University of Southampton, Southampton, United Kingdom; [7]NIHR Southampton Biomedical Research Centre, University Hospital Southampton Foundation NHS Trust, Southampton, United Kingdom; [8]Institute for Research, Development and Innovation, International Medical University, Kuala Lumpur, Malaysia; [9]Department of Biological Sciences, Faculty of Science, Universiti Tunku Abdul Rahman, Kampar, Malaysia.

*Corresponding authors: C.C.Y. – chewchieng@gmail.com; S.C.C. – S.C.Clarke@soton.ac.uk

The Supplementary Data consists of Supplementary Tables S1 – S4, and Supplementary Figures S1 – S3.

Supplementary Table S1. List of the *A. baumannii* reference genomes used to construct the core-genome maximum-likelihood phylogenetic tree presented in **Supplementary Figure S1**. The additional 126 *A. baumannii* genomes from Hospital Sultanah Nur Zahirah (HSNZ), Terengganu, Malaysia that were reported by Din et al. (2025) could be found under BioProject no. PRJNA573295.

ISOLATE	ACCESSION NO.	COUNTRY OF ISOLATION*	PASTEUR MLST**	GLOBAL CLONE***
MRSN23390	VHGI00000000	AFGANISTAN	ST15	GC4
MRSN7735	VHDV00000000	AFGANISTAN	UNK	N/A
Ab825	NTFR00000000	ARGENTINA	ST15	GC4
RBH3	FBXD00000000	AUSTRALIA	ST1	GC1
A85	JACSSO000000000	AUSTRALIA	ST1	GC1
D1279779	AERZ00000000	AUSTRALIA	ST267	N/A
MC1	QXPV00000000	BOLIVIA	ST991	GC7
MC14	QXPO00000000	BOLIVIA	ST25	GC7
MC27	QXPI00000000	BOLIVIA	ST25	GC7
MC31	QXPH00000000	BOLIVIA	ST25	GC7
AB4332	RJLV00000000	BRAZIL	ST78	GC6
AB5375	RJLW00000000	BRAZIL	ST78	GC6
Ab30	VALT00000000	CANADA	ST79	GC5
Canada BC-5	AFDN00000000	CANADA	ST1	GC1
Ab3_Ch	QFLH00000000	CHILE	ST15	GC4
MDR-ZJ06	CP001937	CHINA	ST2	GC2
MDR-TJ	AEOE01000000	CHINA	ST2	GC2
BJAB0715	CP003847	CHINA	ST23	N/A
BJAB07104	CP003846	CHINA	ST2	GC2
BJAB0868	CP003849	CHINA	ST2	GC2
ZW85-1	CP006768	CHINA	ST639	N/A
R2090	LN868200	EGYPT	ST267	N/A
R2091	LN997846	EGYPT	ST126	N/A
AYE	CU459141	FRANCE	ST1	GC1
OIFC098	AMDF00000000	GERMANY	ST10	GC8
A388	CP024418	GREECE	ST1	GC1
MCR6056	NQXL00000000	HONDURAS	ST156	N/A
IS-123	ALII00000000	IRAQ	ST3	GC3
K50	OHJL01000000	KUWAIT	ST158	GC10
AC12	ALAM01000000	MALAYSIA (HSNZ, TERENGGANU)	ST2	GC2
AC29	CP007535	MALAYSIA (HSNZ, TERENGGANU)	ST2	GC2
AC30	ALXD01000000	MALAYSIA (HSNZ, TERENGGANU)	ST2	GC2
CRE1071	SWLT00000000	MALAYSIA (KELANTAN)	ST2	GC2
CRE245	SWLP00000000	MALAYSIA (KUALA LUMPUR)	ST2	GC2
CRE341	SWLO00000000	MALAYSIA (KUALA LUMPUR)	ST25	GC7
CRE596	SWLL00000000	MALAYSIA (KUALA LUMPUR)	ST2	GC2
CRE648	SWLJ00000000	MALAYSIA (KUALA LUMPUR)	ST2	GC2

CRE85	SWLI00000000	MALAYSIA (PAHANG)	ST164	GC11
CRE157	SWLR00000000	MALAYSIA (PERAK)	ST2	GC2
CRE158	SWLQ00000000	MALAYSIA (PERAK)	ST2	GC2
CRE400	SWLN00000000	MALAYSIA (PERAK)	ST2	GC2
CRE449	SWLM00000000	MALAYSIA (PAHANG)	ST2	GC2
CRE645	SWLK00000000	MALAYSIA (PERAK)	ST2	GC2
CRE98	SWLH00000000	MALAYSIA (SARAWAK)	ST2	GC2
Cab65	CP060994	MALAYSIA (SEGAMAT, JOHOR)	ST142	N/A
C-15	JAPCWU000000000	MALAYSIA (SEGAMAT, JOHOR)	ST216	N/A
C-28	JANEYD000000000	MALAYSIA (SEGAMAT, JOHOR)	ST284	N/A
C-39	JANEYC000000000	MALAYSIA (SEGAMAT, JOHOR)	ST338	N/A
C-55	JAPCWT000000000	MALAYSIA (SEGAMAT, JOHOR)	ST336	N/A
C-59	JAPCWS000000000	MALAYSIA (SEGAMAT, JOHOR)	UNK	N/A
C-61	JANEYB000000000	MALAYSIA (SEGAMAT, JOHOR)	ST1411	N/A
C-64	JAPCWR000000000	MALAYSIA (SEGAMAT, JOHOR)	ST331	N/A
C-72	JAPCWQ000000000	MALAYSIA (SEGAMAT, JOHOR)	ST49	N/A
C-95	JAPCWP000000000	MALAYSIA (SEGAMAT, JOHOR)	UNK	N/A
C-98	JANEYA000000000	MALAYSIA (SEGAMAT, JOHOR)	ST1	GC1
C-102	JANEXZ000000000	MALAYSIA (SEGAMAT, JOHOR)	ST2108	N/A
H-6657	JAPCWO000000000	MALAYSIA (SEGAMAT, JOHOR)	ST2	GC2
H-6668	JANEXY000000000	MALAYSIA (SEGAMAT, JOHOR)	ST459	N/A
H-7940	JAPCWN000000000	MALAYSIA (SEGAMAT, JOHOR)	ST2	GC2
H-10112	JANEXX000000000	MALAYSIA (SEGAMAT, JOHOR)	ST2	GC2
H-10156	JAPCWM000000000	MALAYSIA (SEGAMAT, JOHOR)	ST2	GC2
H-10299	JANEXW000000000	MALAYSIA (SEGAMAT, JOHOR)	ST1566	N/A
H-10858	JAPCWL000000000	MALAYSIA (SEGAMAT, JOHOR)	ST2	GC2
H-11553	JANEXV000000000	MALAYSIA (SEGAMAT, JOHOR)	ST10	GC8
H-11699	JANEXU000000000	MALAYSIA (SEGAMAT, JOHOR)	ST2	GC2
H-52446	JAPCWK000000000	MALAYSIA (SEGAMAT, JOHOR)	ST2	GC2
H-79532	JAPCWJ000000000	MALAYSIA (SEGAMAT, JOHOR)	ST23	N/A
H-80330	JAPCWI000000000	MALAYSIA (SEGAMAT, JOHOR)	ST2	GC2
H-80359	JANEXT000000000	MALAYSIA (SEGAMAT, JOHOR)	ST2	GC2
H-80361	JAPCWH000000000	MALAYSIA (SEGAMAT, JOHOR)	ST2	GC2
H-80400	JAPCWG000000000	MALAYSIA (SEGAMAT, JOHOR)	ST2	GC2

CRE1159	SWLS00000000	MALAYSIA (SELANGOR)	ST2	GC2
10042	CP023026	MEXICO	ST2	GC2
5845	CP023034	MEXICO	ST2	GC2
AF-401	CP018254	MEXICO	ST79	GC5
9102	CP023029	MEXICO	ST1	GC1
810CP	CP026338	MEXICO CITY	ST156	N/A
ACICU	CP031380	ROME	ST2	GC2
1656-2	CP001921	SOUTH KOREA	ST2	GC2
TCDC-AB0715	CP002522	TAIWAN	ST2	GC2
TYTH-1	CP003856	TAIWAN	ST2	GC2
VNMU 136	WYAB00000000	UKRINE	ST78	GC6
ATCC19606	CZWC00000000	USA	ST52	N/A
NIPH 1734	APOX00000000	USA	ST15	GC4
OIFC137	AFDK00000000	USA	ST3	GC3
Naval-81	AFDB00000000	USA	ST3	GC3
WC-A-694	AMTA00000000	USA	ST3	GC3
AB307-0294	CP001172	USA	ST1	GC1
AB0057	ABJM01000000	USA	ST1	GC1
PR371	NGCY00000000	USA	ST78	GC6
TG29392	RFEB01000000	USA	ST78	GC6
TG31986	RFBD01000000	USA	ST78	GC6
LAC-4	JICJ00000000	USA	ST10	GC8
SDF	CU468230	UNKNOWN	ST17	N/A
CIP70.10	LN865143	UNKNOWN	ST126	N/A
ATCC17978	CACVBA000000000	UNKNOWN	ST437	N/A

*The town or state in Malaysia where the isolates were obtained are indicated in parentheses.
**UNK = unknown
***N/A = not available

Supplementary Table S2. Pairwise SNP distances between the core regions of the *bla*$_{NDM-1}$-encoded plasmids with pAC1530 from *A. nosocomialis* AC1530 as reference plasmid, as determined using snp-dists v0.8.2

	pAC1530 (Reference)	pAC1633-1	pAC1839-1	pAC1932-1	pAC2013-1	pAC2014-1
pAC1530 (Reference)	0	2	85	200	79	84
pAC1633-1	2	0	85	200	79	84
pAC1839-1	85	85	0	129	8	13
pAC1932-1	200	200	129	0	121	126
pAC2013-1	79	79	8	121	0	5
pAC2014-1	84	84	13	126	5	0

Supplementary Table S3. Estimated copy number of the *bla*$_{NDM-1}$-encoded plasmids identified in this study. The coverage depth of the assembled short-read contigs which mapped to the complete plasmid sequences (that was obtained through hybrid assembly of both long- and short-reads) was compared relative to the chromosome (taken as the longest assembled contig, i.e., contig_1).

																	**Average**
**AC1839**	contig_1	contig_11	contig_13	contig_14	contig_18	contig_20	contig_21	contig_22	contig_23	contig_25	contig_27	contig_28	contig_30	contig_32	contig_33	contig_39	
coverage	285.2	338.5	349.5	348.5	310.7	334.2	330.9	310.5	346.2	669.7	648.3	669.6	648.2	312.7	613.6	512.4	
copy number	1.0	1.2	1.2	1.2	1.1	1.2	1.2	1.1	1.2	2.3	2.3	2.3	2.3	1.1	2.2	1.8	**1.6**
**AC1932**	contig_1	contig_20	contig_31	contig_68	contig_79	contig_81	contig_91	contig_94	contig_96	contig_110	contig_127	contig_140	contig_147	contig_152			
coverage	312.3	316.9	334.5	312.4	206.2	245.1	308.5	314.3	322.1	268.4	520.8	648.8	1815.7	151.3			
copy number	1.0	1.0	1.1	1.0	0.7	0.8	1.0	1.0	1.0	0.9	1.7	2.1	5.8	0.5			**1.4**
**AC2013**	contig_1	contig_16	contig_23	contig_34	contig_38	contig_42	contig_45	contig_46	contig_48	contig_50	contig_53	contig_57	contig_58				
coverage	305.3	375.9	373.7	377.4	276.1	397.8	303.9	391.4	342.6	644.2	582.5	646.6	458.4				
copy number	1.0	1.2	1.2	1.2	0.9	1.3	1.0	1.3	1.1	2.1	1.9	2.1	1.5				**1.4**
**AC2014**	contig_1	contig_23	contig_32	contig_55	contig_59	contig_84	contig_98	contig_99	contig_115	contig_130	contig_131	contig_132	contig_136	contig_149	contig_152		
coverage	293.7	284.1	265.5	240.6	264.3	164.9	253	304.1	192.6	464.6	635.6	2623.4	467.4	616.6	180.4		
copy number	1.0	1.0	0.9	0.8	0.9	0.6	0.9	1.0	0.7	1.6	2.2	8.9	1.6	2.1	0.6		**1.7**

Supplementary Table S4. The organization, orientation and nucleotide sequences of the *xrs* (or p*dif*) sites in the *bla*$_{OXA-58}$-harbouring-plasmids. Nucleotide differences are highlighted in yellow. The 6 bp spacer region in between the XerC/XerD sites are underlined.

Name	Plasmid	*xrs* (or p*dif*) sequence	Site	Position in plasmid (nt)
*xrs*1	pOXA58-AP882	ATTTCGTATAA GGTGTA TTATGTTAATT	C|D	23666-23693
	pAC1530	ATTTCGTATAA GGTGTA TTATGTTAATT		152111-152138
	pAC1633-1	ATTTCGTATAA GGTGTA TTATGTTAATT		153651-153678
	pAC1839-1	ATTTCGTATAA GGTGTA TTATGTTAATT		151059-151,086
*xrs*2	pOXA58-AP882	ATTTAACATAA TGGTGT TATACGAAATT	D|C	25138-25165
	pAC1530	ATTTAACATAA TGGTGT TATACGAAATT		153583-153610
	pAC1633-1	ATTTAACATAA TGGTGT TATACGAAATT		155123-155150
	pAC1839-1	ATTTAACATAA TGGTGT TATACGAAATT		152531-152558
*xrs*3	pOXA58-AP882	GATTCGTATAA GGTGTA TTATGTTAATT	C|D	25875-25902
	pAC1530_	GATTCGTATAA GGTGTA TTATGTTAATT		154320-154347
	pAC1633-1	GATTCGTATAA GGTGTA TTATGTTAATT		155860-155887
	pAC1839-1	GATTCGTATAA GGTGTA TTATGTTAATT		153268-153295
	pAC2013-1	ATTTCGTATAA GGTGTA TTATGTTAATT		152345-152372
	pAC2014-1	ATTTCGTATAA GGTGTA TTATGTTAATT		154761-154788
*xrs*4	pOXA58-AP882	ATTTAACATAA TGGCTG TTATACGAAAC	D|C	28005-28032
	pAC1530	ATTTAACATAA TGGCTG TTATACGAAAC		156450-156477
	pAC1633-1	ATTTAACATAA TGGCTG TTATACGAAAC		157990-158017
	pAC1839-1	ATTTAACATAA TGGCTG TTATACGAAAC		155398-155425
	pAC2013-1	ATTTAACATAA TGGCTG TTATACGAAAC		154475-154502
	pAC2014-1	ATTTAACATAA TGGCTG TTATGCGAAAC		156891-156918
*xrs*5	pOXA58-AP882	ATTTTGTATAA GGTGTA TTATGTTAATT	C|D	31399-31426
	pAC1530	ATTTTGTATAA GGTGTA TTATGTTAATT		159844-159871
	pAC1633-1	ATTTTGTATAA GGTGTA TTATGTTAATT		161384-161411
	pAC1839-1	ATTTTGTATAA GGTGTA TTATGTTAATT		160280-160307
	pAC2013-1	ATTTTGTATAA GGTGTA TTATGTTAATT		157869-157896
*xrs*6	pOXA58-AP882	ATTTAACATAA TGGGCG TTATACGAAAT	D|C	32966-32993
	pAC1530	ATTTAACATAA TGGGCG TTATGCGAAGT		161411-161438
	pAC1633-1	ATTTAACATAA TGGGCG TTATGCGAAGT		162951-162978
	pAC1839-1	ATTTAACATAA TGGGCG TTATACGAAAT		161847-161874
	pAC2013-1	ATTTAACATAA TGGGCG TTATACGAAAT		159436-159463
*xrs*7	pOXA58-AP882	ACTTCGCATAA CGCCCA TTATGTTAATT	C|D	33820-33847
	pAC1530	ATTTCGTATAA CGCCCA TTATGTTAATT		162265-162292
	pAC1633-1	ATTTCGTATAA CGCCCA TTATGTTAATT		163805-163832
	pAC1839-1	ACTTCGCATAA CGCCCA TTATGTTAATT		164188-164215
	pAC2013-1	ACTTCGCATAA CGCCCA TTATGTTAATT		160290-160317
*xrs*8	pOXA58-AP882	ACTTAACATAA TGGCGG TTATACGAAAT	D|C	34462-34489
	pAC1530	ACTTAACATAA TGGCGG TTATACGAAAT		162907-162934
	pAC1839-1	ACTTAACATAA TGGCGG TTATACGAAAT		164830-164857
	pAC2013-1	ACTTAACATAA TGGCGG TTATACGAAAT		160932-160959
*xrs*9	pOXA58-AP882	ATTTCGTATAA GGTGTA TTATGTTAATT	C|D	35186-35213
	pAC1530	ATTTCGTATAA GGTGTA TTATGTTAATT		163631-163658
	pAC1839-1	ATTTCGTATAA GGTGTA TTATGTTAATT		165554-165581
	pAC2013-1	ATTTCGTATAA GGTGTA TTATGTTAATT		161656-161683

Name	Plasmid	p*dif* sequence	Site	Position in plasmid (nt)
*xrs*10	pOXA58-AP882	ATTTAACATAA TGGCTG TTATGCGAAAC	D|C	35681-35708
	pAC1530	ATTTAACATAA TGGCTG TTATGCGAAAC		164126-164153
	pAC1633-1	ACTTAACATAA TGGCTG TTATGCGAAAC		164447-164474
	pAC1839-1	ATTTAACATAA TGGCTG TTATGCGAAAC		166049-166076
	pAC2013-1	ATTTAACATAA TGGCTG TTATGCGAAAC		162151-162178
*xrs*11	pOXA58-AP882	ATTTCGTATAA GGTGTA TTATGTTAATT	C|D	36412-36439
	pAC1530	ATTTCGTATAA GGTGTA TTATGTTAATT		164857-164884
	pAC1633-1	ATTTCGTATAA GGTGTA TTATGTTAATT		165178-165205
	pAC1839-1	ATTTCGTATAA GGTGTA TTATGTTAATT		166780-166807
	pAC1932-1	ATTTCGTATAA GGTGTA TTATGTTAATT		156039-156066
	pAC2013-1	ATTTCGTATAA GGTGTA TTATGTTAATT		162882-162909
	pAC2014-1	ATTTCGTATAA GGTGTA TTATGTTAATT		158903-158930
*xrs*12	pOXA58-AP882	ATTTAACATAA AATTTC TTATGTGAAGT	D|C	2528-2555
	pAC1530	ATTTAACATAA AATTTC TTATGTGAAGT		167835-167862
	pAC1633-1	ATTTAACATAA AATTTC TTATGTGAAGT		168156-168183
	pAC1839-1	ATTTAACATAA AATTTC TTATGTGAAGT		169758-169785
	pAC1932-1	ATTTAACATAA AATTTC TTATGTGAAGT		159017-159044
	pAC2013-1	ATTTAACATAA AATTTC TTATGTGAAGT		165860-165887
	pAC2014-1	ATTTAACATAA AATTTC TTATGTGAAGT		161881-161908

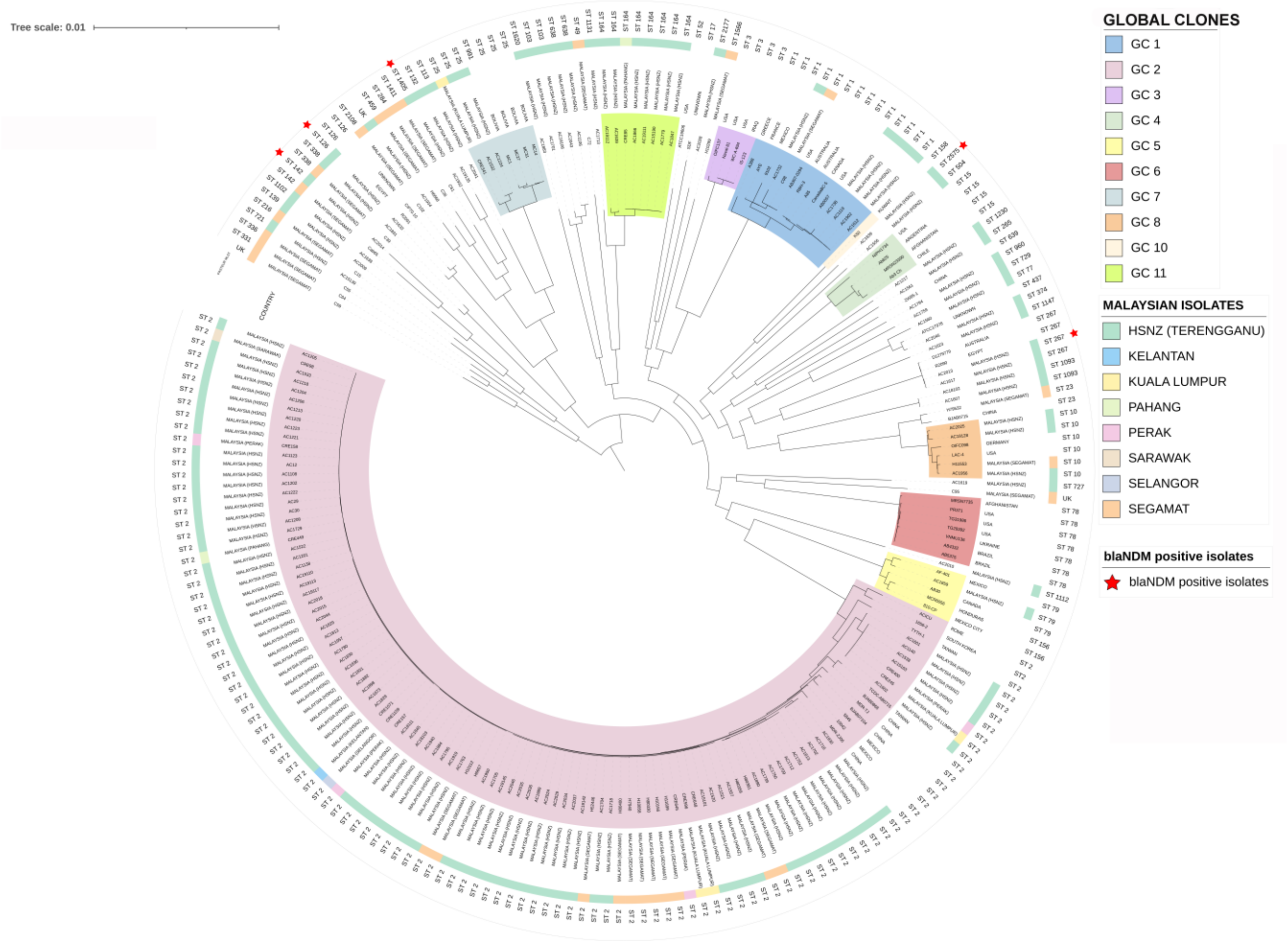

Supplementary Figure S1. Midpoint-rooted core-genome maximum-likelihood phylogenetic tree of Malaysian *A. baumannii* isolates as presented in Din et al. (2025) (1) along with several reference *A. baumannii* genomes that represent the major Global Clones (GCs) 1 – 11 (listed in **Supplementary Table S1**), which were highlighted as indicated in the upper right legend. The outer coloured ring depicted the states in Malaysia from which the isolates were obtained. The $ST_{Pasteur}$ of the isolates were also depicted in the outermost ring and isolates harbouring the *bla*$_{NDM-1}$-encoded plasmid presented in this study were marked with a red-coloured star symbol. A 720 dpi version of this Figure could be seen in Figshare at: 10.6084/m9.figshare.30744803

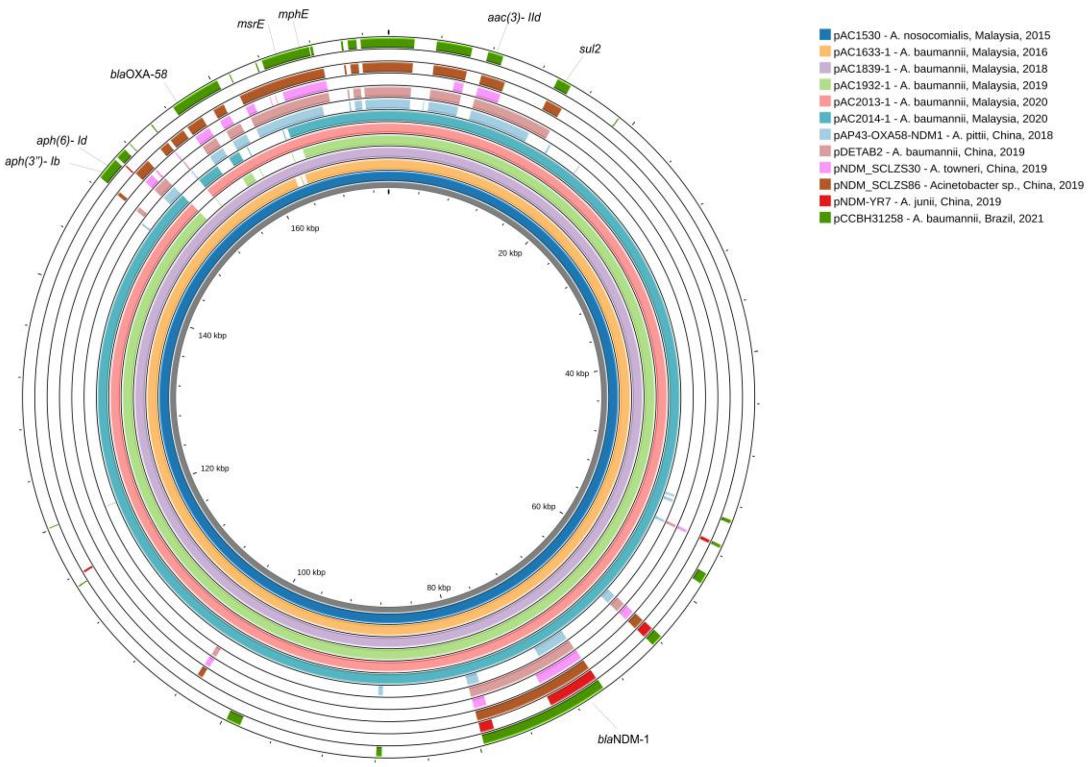

Supplementary Figure S2. Comparison of the *bla*$_{\mathrm{NDM-1}}$-encoded plasmids identified in this study (pAC1839-1, pAC1932-1, pAC2013-1, and pAC2014-1), our earlier study (pAC1530 and pAC1633-1) (2) and other *bla*$_{\mathrm{NDM-1}}$-encoded plasmids in the literature with the relevant antibiotic resistance genes indicated. The other plasmid sequences used in this CGView/Proksee (3) comparison are: pAP43-OXA58-NDM1 (accession no. CP043053.1), pDETAB2 (CP047975.1) (4), pNDM_SCLZS30 (CP090384.1) (5), pNDM_SCLZS86 (CP090865.1) (6), pCCBH31258 (CP101888.1) (7), and pNDM-YR7 (CP059559.1) (8).

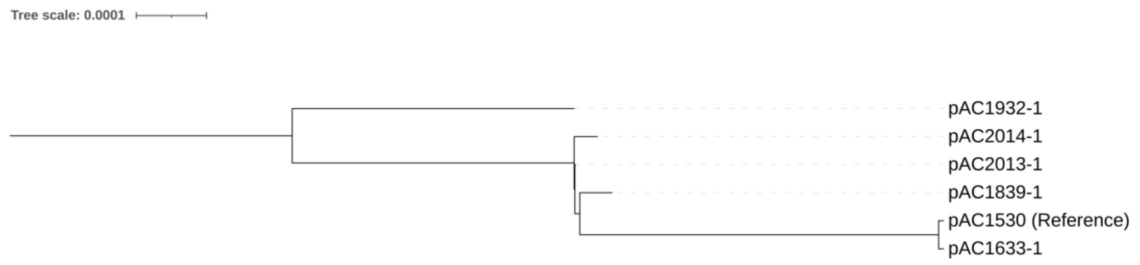

Supplementary Figure S3. Maximum likelihood phylogenetic tree generated from the pairwise SNP distance of the core regions of the large bla_{NDM-1}-encoded plasmids using pAC1530 from *A. nosocomialis* AC1530 (2) as the reference.

References cited in the Supplementary Data:

1. Din NS, Mohd. Rani F, Alattraqchi AG, Ismail S, A. Rahman NI, Cleary DW, et al. Whole-genome sequencing of *Acinetobacter baumannii* clinical isolates from a tertiary hospital in Terengganu, Malaysia (2011–2020), revealed the predominance of the Global Clone 2 lineage. *Microb Genom*. 2025;11(2):001345. doi:10.1099/mgen.0.001345

2. Alattraqchi AG, Mohd Rani F, A. Rahman NI, Ismail S, Cleary DW, Clarke SC, Yeo CC. Complete genome sequencing of *Acinetobacter baumannii* AC1633 and *Acinetobacter nosocomialis* AC1530 unveils a large multidrug-resistant plasmid encoding the NDM-1 and OXA-58 carbapenemases. *mSphere*. 2021;6(1):e01076-20. doi: 10.1128/mSphere.01076-20.

3. Grant JR, Enns E, Marinier E, Mandal A, Herman EK, Chen CY, Graham M, Van Domselaar G, Stothard P. Proksee: in-depth characterization and visualization of bacterial genomes. *Nucleic Acids Res.* 2023;51(W1):W484-W492. doi: 10.1093/nar/gkad326.

4. Liu H, Moran RA, Chen Y, Doughty EL, Hua X, Jiang Y, Xu Q, Zhang L, Blair JM, McNally A, van Schaik W. Transferable *Acinetobacter baumannii* plasmid pDETAB2 encodes OXA-58 and NDM-1 and represents a new class of antibiotic resistance plasmids. *J Antimicrob Chemothe*r. 2021;76(5):1130-1134. doi: 10.1093/jac/dkab005.

5. Li Y, Qiu Y, Fang C, Dai X, Zhang L. Coexistence of *bla*OXA-58 and *bla*NDM-1 on a novel plasmid of GR59 from an *Acinetobacter towneri* isolate. *Antimicrob Agents Chemother*. 2022;66(6):58-60. doi: 10.1128/aac.00206-22.

6. Li Y, Qiu Y, Fang C, Tang M, Dai X, Zhang L. Characterisation of a novel GR31 plasmid co-harbouring *bla*NDM-1 and *bla*OXA-58 in an *Acinetobacter* sp. isolate. *J Glob Antimicrob Resist.* 2022;29:212-214. doi: 10.1016/j.jgar.2022.03.015.

7. Rodrigues DC, Silveira MC, Pribul BR, Karam BR, Picão RC, Kraychete GB, Pereira FM, de Lima RM, de Souza AK, Leão RS, Marques EA. Genomic study of *Acinetobacter baumannii* strains co-harboring *bla*OXA-58 and *bla*NDM-1 reveals a large multidrug-resistant plasmid encoding these carbapenemases in Brazil. *Front Microbiol*. 2024;15:1439373. doi: 10.3389/fmicb.2024.1439373.

8. Tang B, Wang C, Sun D, Lin H, Ma J, Guo H, Yang H, Li X. In silico characterization of *bla*NDM-harboring conjugative plasmids in *Acinetobacter* species. *Microbiol Spectr*. 2022;10(6):e02102-22. doi: 10.1128/spectrum.02102-22.
